# Does tolerance to ethanol-induced ataxia explain the sensitized response to ethanol?

**DOI:** 10.3389/fpsyt.2024.1418490

**Published:** 2024-08-30

**Authors:** Cheryl Reed, Tamara J. Phillips

**Affiliations:** ^1^ Department of Behavioral Neuroscience, Oregon Health & Science University, Portland, OR, United States; ^2^ Portland Alcohol Research Center, Oregon Health & Science University, Portland, OR, United States; ^3^ Veterans Affairs Portland Health Care System, Portland, OR, United States

**Keywords:** sensitization, tolerance, locomotor activity, ataxia, ethanol, mouse

## Abstract

Under conditions of repeated exposure to ethanol, a sensitized locomotor stimulant response develops in some strains of mice. It has been hypothesized that the sensitized response is a consequence of tolerance development to the sedative/incoordinating effects of ethanol. Conversely, ethanol-induced sensitization and tolerance may be independent effects of repeated ethanol exposure. A published study in C57BL/6J by DBA/2J recombinant inbred strains concluded that the two phenomena are not genetically related and thus perhaps mechanistically distinct. To extend evaluation beyond the genetic variance found in C57BL/6J and DBA/2J mice and examine phenotypic associations, we simultaneously measured ethanol-induced sensitization and tolerance in a genetically diverse panel of 15 standard inbred mouse strains and a genetically heterogeneous stock that was produced by the intercrossing of eight inbred mouse strains. Changes in activity counts and ataxia ratio across repeated ethanol treatments indexed sensitization and tolerance, respectively. Photocell beam breaks provided the measure of activity, and foot slip errors corrected for activity in a grid test provided a measure of coordination. The results were strain and individual dependent. The genetic correlation between magnitude of sensitization and tolerance was not significant in the panel of inbred strains, but when individual data were correlated, without regard to strain, there was a significant correlation. This relationship was also significant in the genetically heterogeneous population of mice. However, magnitude of tolerance explained only 10% of the variance in sensitization among individuals of the inbred strain population, whereas it explained 44% of the variance among individuals of the eight-strain cross. When repeated exposures to ethanol were disassociated from the test apparatus, this relationship in the eight-strain cross disappeared. Furthermore, days to peak sensitization and tolerance across days did not perfectly mirror each other. Overall, our data do not support shared genetic mechanisms in sensitization and tolerance development but suggest a partial relationship among individuals that could be related to drug–environment associations.

## 1 Introduction

Procedures for measuring behavioral stimulant and sedative-ataxic effects of alcohol (ethanol; EtOH) in mice were established decades ago to model similar effects observed in humans. Sensitivity to stimulant and the sedative effects of alcohol have some value in predicting risk for alcohol use in humans (1–6). In mice, these effects are most often measured by examining EtOH-induced changes in locomotor activity level and motor coordination (7–13). With repeated exposure to EtOH, alterations in initial responses often occur (14). In some genotypes of mice, initial locomotor stimulant response to EtOH increases, a phenomenon termed sensitization (11, 15–22). Tolerance, or a decrease in the initial ataxic or sedative response to EtOH, is a common result of repeated EtOH exposure (21, 23–27). These opposite directional outcomes, increased activity in concert with decreased ataxia, have led some to suggest that EtOH-induced locomotor sensitization is a by-product of tolerance development to the sedative–ataxic effects of EtOH, although thus far the hypothesis has received little investigation (17, 21, 26, 28). This question is important because it addresses whether EtOH-induced tolerance and sensitization should be studied as related or independent behavioral adaptations, with related or independent underlying neural mechanisms.

A relationship between EtOH-induced tolerance and sensitization can be examined at the phenotypic and genetic levels. In a previous study, we examined the genetic association between EtOH tolerance and sensitization in a panel of 24 recombinant inbred (RI) mouse strains derived from two inbred strain progenitors, C57BL/6J and DBA/2J (21). We surmised that if tolerance to the ataxic effects of EtOH underlies the development of sensitization, then the strains that develop the most tolerance should also develop relatively more sensitization. A strong strain mean correlation between magnitude of tolerance and sensitization would indicate that some of the same genes impact the two traits. That experiment failed to support the hypothesis of genetic parity. Thus, the strain distribution patterns for the two traits were different; strains that developed the most tolerance were not necessarily the ones that developed the most sensitization. However, the conclusion was limited to genetic correlation and genetic variation existing between the C57BL/6J and DBA/2J strains from which the RI strains were derived. Therefore, in the current studies, we extend the question to a larger gene pool and assess both genetic and phenotypic correlation, the latter of which can be impacted by both genetic and environmental influences and gets at the question of individual variation.

All studies described here address the hypothesis that magnitude of behavioral sensitization is related to magnitude of behavioral tolerance to EtOH. In experiment 1, the question of genetic correlation was addressed in a genetically diverse panel of 15 inbred mouse strains; phenotypic correlations were also examined by considering individual differences without regard to strain. In experiment 2, the relationship between tolerance and sensitization was further assessed in the DBA/2J inbred mouse strain, based on existing evidence supported in experiment 1, for strong EtOH-induced sensitization in this strain (19, 29–35). DBA/2J mice also develop robust behavioral tolerance to acute and chronic EtOH (36–38). In experiments 3 and 4, a genetically heterogeneous stock (HS) of mice allowed us to further examine phenotypic correlations between traits. These mice are the product of an eight-way cross of a diverse set of inbred mouse strains (39, 40). They are from a single breeding population, housed in a single colony under routine environmental conditions throughout rearing and thereafter. Although the inbred strains were housed under the same conditions, once they arrived in Portland, colony rooms and perhaps other environmental conditions at the source varied among the strains that could have impacted phenotypic correlations. Therefore, we wanted to determine whether a similar outcome would be obtained for the collection of inbred strains and the strain intercross population. In all experiments, we phenotyped the mice for EtOH-induced locomotor stimulation and sensitization, while simultaneously measuring grid-test ataxia and tolerance, to determine whether levels of any of the traits were predictive of or corresponded with levels of the other traits. We hypothesized that the patterns of sensitization and tolerance development across days should be coordinated, indicating that a change in one phenotype is necessary for a change in the other to occur.

## 2 Materials and methods

All experiments included adult male mice. For the genetic models, it was necessary to test a large number of mice in order to test the association hypothesis. Resources were not available to test enough male and female mice to study potential sex differences, which were not the focus of the current studies but should be considered in future investigations. Ages and group sizes for each study are given with the experimental details. For experiment 1, the mice were from 15 standard inbred strains (129/J, A/HeJ, AKR/J, BALB/cJ, C3H/HeJ, C57BL/6J, C57BR/CdJ, C57L/J, CBA/J, CE/J, DBA/1J, DBA/2J, PL/J, SJL/J, and SWR/J). We chose these mouse strains for their genetic diversity and because they have been characterized for several other EtOH-related phenotypes (41–46). Experiment 2 tested only DBA/2J mice. Withdrawal seizure control (WSC-1 and WSC-2) mice were tested in experiments 3 and 4. The WSC lines are comparable to the HS/Ibg derived by McClearn and Kakihana (40). They were created as non-selectively bred heterogeneous controls for lines bred for high- and low-EtOH withdrawal seizure susceptibility (39). The WSC breeding colonies were established within the veterinary medical unit (VMU) of the Veterans Affairs Portland Health Care System (VAPORHCS) from HS/Ibg breeders and are the product of an eight-way cross of the inbred strains: A, AKR, BALB/c, C3H/2, C57BL, DBA/2, IS/Bi, and RIII; strains IS/Bi and RIII no longer exist. We have previously shown that the WSC mice display robust EtOH-induced sensitization (47, 48), which is why they were utilized in these studies.

All inbred strain mice were purchased from The Jackson Laboratory (Bar Harbor, ME), shipped to the VAPORHCS, and maintained in the VMU in groups of three to five per cage with same-strain cage mates. The mice had free access to water and standard laboratory chow (Purina Laboratory Rodent Chow 5001; Purina Mills, St. Louis, MO, USA), except during testing. Caging was made of standard polycarbonate mouse cages (28 × 18 × 13 cm) lined with corncob bedding. Animals shipped to the VMU were allowed a 2-week acclimation period prior to testing. WSC mice were produced by breeding pairs within the VMU and, once weaned, were housed in same-sex cages of two to four mice. The caging details were identical to those described for the inbred strains.

Ambient temperature averaged 21°C ± 2°C, and fluorescent ceiling lights were on for 12 h each day (lights on between 0600 and 1800 h). The animals were tested between 0800 h and 1600 h. All procedures using animals were approved by the VAPORHCS Institutional Animal Care and Use Committee and were performed in accordance with the National Institutes of Health Guidelines for the Care and Use of Laboratory Animals (49).

### 2.1 Grid test apparatus and locomotor activity testing

The grid test apparatus used for assessing ataxia has been described previously (21). The test apparatus comprises a clear acrylic plastic box (15 × 15 × 20 cm; w × d × h), with a removable acrylic plastic lid and no bottom. The box is placed on 1.25-cm^2^ carpenter’s cloth grid, suspended 1.25 cm above a stainless steel plate. An acrylic plastic frame separates the grid and the plate, with a connection established via a contact relay (Med Associates, St. Albans, VT, USA) when the mouse’s foot slips off the grid and contacts the underlying plate. This contact is recorded by a computer as a foot slip error. Four grid test devices were utilized for this research, each positioned in the center of an automated activity monitor (Omnitech; Columbus, OH; 40 × 40 × 30 cm), oriented so that two photocell beams opposite receptors transected the acrylic box on each side at approximately 5 cm intervals. Interruption of the photocell beams was automatically recorded and provided a measure of locomotor activity. Before the start of each test session, the location of the grid test apparatus within the activity monitor was confirmed to ensure that no photocell beams were impeded by the grid test apparatus. This setup of the grid test apparatus within the locomotor activity chamber allowed for the simultaneous measurement of foot slip errors and locomotor activity. The ratio of foot slip errors to activity counts was calculated to provide a measure of ataxia that corrects for variability in locomotor activity; we refer to this measure as the ataxia ratio (7, 21).

### 2.2 Drugs

EtOH was purchased from Pharmco Products (Brookfield, CT, USA) and diluted with 0.9% saline (Baxter Healthcare Crop, Deerfield, IL, USA) to create a 20% (v/v) EtOH solution.

### 2.3 Blood EtOH concentration determination

Blood samples (20 µL) from the retro-orbital sinus were analyzed for blood EtOH concentration (BEC) in comparison to a standard curve using a gas chromatograph (Hewlett-Packard, model number; Palo Alto, CA, USA) with flame ionization detection and our previously published procedures (50). After each sample was collected, it was placed in 50 µL of ice-cold 5% zinc sulfate solution, and then a 50-µL aliquot of 0.3 N barium hydroxide solution and a 300-µL aliquot of distilled deionized water were added. After centrifugation, the supernatant was removed and used in the gas chromatography analysis, with values expressed in mg/mL.

### 2.4 Experimental procedures

For all studies, the mice were moved into the testing room for an acclimation period of 60 min prior to testing. All injections were delivered intraperitoneally (IP) at 10 mL/kg injection volume, and the side of the abdomen was alternated with each injection to reduce discomfort. All tests began immediately after injection of saline or EtOH, and data were collected in 5-min time periods.

#### 2.4.1 Experiment 1: acute and repeated EtOH effects on locomotor activity and coordination in 15 inbred mouse strains

The mice were tested using an experimental protocol that we designed to measure initial EtOH response, followed by change in response after repeated EtOH administration to determine the magnitude of EtOH-induced locomotor sensitization. We employed this procedure in our previous study to evaluate the genetic association between EtOH-induced tolerance and sensitization in a panel of RI mouse strains (21). A 2-g/kg EtOH dose was used; doses of 2–2.5 g/kg are typical for studies across multiple labs in examinations of EtOH-induced sensitization [reviewed in (51)]. The study schedule is summarized in **Table 1**. The chronic saline (CS) group received saline before placement in the grid test apparatus on all days, except on day 11 when they received EtOH. The chronic EtOH (CE) group received EtOH on all test days (at a 48-h interval), except on days 1 and 2 when they received saline. Day 1 served as the habituation day, and day 2 provided baseline activity and coordination data for both groups. Day 3 provided a measure of initial response to EtOH in the CE group; days 5, 7, 9, and 11 provided measurements from which a change in EtOH response could be determined, reflecting the development of sensitization to the initial stimulant effect and tolerance to the initial ataxic effect of EtOH in the CE group. Day 11 provided a measure of initial response to EtOH in the CS group and a between-groups measure of sensitization/tolerance when compared to the day 11 CE group data. No treatment or testing occurred on days 4, 6, 8, or 10; the animals were left undisturbed in their cages in the colony room. All test sessions were 10 min in duration; we and others have previously documented that EtOH has robust stimulant and ataxic effects during the initial 5–15 min post-administration (52–56). The total number of mice tested was 295; however, data for four mice were not included in the final analysis due to computer-related failures that occurred during data collection. Therefore, data from 291 mice were included in the final analysis, resulting in a group size of nine to 10 mice per strain per treatment group. The average age of the mice at the initiation of testing was 83 ± 1 days, with a range of 59 to 101 days.

**Table 1:** Protocol for testing acute and repeated EtOH effects on locomotor activity and coordination in 15 inbred mouse strains.

	Days										
	1	2	3	4	5	6	7	8	9	10	11
CS group											
**Injection**	SAL	SAL	SAL	NONE	SAL	NONE	SAL	NONE	SAL	NONE	EtOH
**Test?**	Y	Y	Y	N	Y	N	Y	N	Y	N	Y
CE group											
**Injection**	SAL	SAL	EtOH	NONE	EtOH	NONE	EtOH	NONE	EtOH	NONE	EtOH
**Test?**	Y	Y	Y	N	Y	N	Y	N	Y	N	Y

CS group mice were tested after IP saline injection on all test days except day 11. CE group mice were tested after IP injection of 2 g/kg EtOH on all test days except days 1 and 2, when they received saline. Tests were 10 min in duration and began immediately after injection. No treatment or testing occurred on days 4, 6, 8, or 10.

SAL, saline; NONE, no treatment; EtOH, ethanol; Y, mice were tested; N, mice were not tested.

#### 2.4.2 Experiment 2: acute and repeated EtOH effects on locomotor activity and coordination in DBA/2J mice

The study schedule is summarized in **Table 2** and was similar to that for experiment 1; however, EtOH treatments were administered daily, rather than every other day. Comparisons across studies suggested that daily EtOH treatments induced greater sensitization (e.g., 52) than did treatments given every other day (e.g., 21) in DBA/2J mice. Furthermore, we increased the dose of EtOH to 2.5 g/kg based on evidence that this dose induces more robust EtOH-induced sensitization in DBA/2J mice (29, 32, 34). The CS group mice received saline on all days, except on day 15 when they received 2.5 g/kg EtOH. The CE group mice received 2.5 g/kg EtOH on all days, except on days 1 and 2 when they received saline. Day 1 served as the habituation day; day 2 provided baseline activity and coordination data; day 3 provided a measure of initial response to EtOH in the CE group; days 6, 9, 12, and 15 provided measurements from which sensitization/tolerance could be determined in the CE group; and day 15 provided a measure of initial EtOH response in the CS group mice and a between-groups measure of sensitization/tolerance. The test day data analyzed were for the first 10 min after treatment, as for experiment 1. On days between test days, the mice were treated with saline or EtOH in their colony room and immediately returned to their home cages after injection. A total of 24 DBA/2J mice were tested; however, a total of eight mice, four from each treatment group, were discarded due to issues experienced upon collecting foot slip data on one or more days of testing; the final group size was eight mice per treatment group. All mice were born on the same day and were 57 days old at the initiation of testing.

**Table 2:** Protocol for testing acute and repeated EtOH effects on locomotor activity and coordination in DBA/2J and WSC mice.

Days											
	1	2	3	4–5	6	7–8	9	10–11	12	13–14	15
CS group											
**Injection**	SAL	SAL	SAL	SAL	SAL	SAL	SAL	SAL	SAL	SAL	EtOH
**Test?**	Y	Y	Y	N	Y	N	Y	N	Y	N	Y
CE group											
**Injection**	SAL	SAL	EtOH	EtOH	EtOH	EtOH	EtOH	EtOH	EtOH	EtOH	EtOH
**Test?**	Y	Y	Y	N	Y	N	Y	N	Y	N	Y

CS group mice were tested after IP saline injection on all test days except day 11. CE group mice were tested after IP injection of 2.5 g/kg EtOH on all test days except days 1 and 2, when they were administered saline.

SAL, saline; EtOH, ethanol; Y, mice were tested; N, mice were not tested.

#### 2.4.3 Experiment 3: acute and daily repeated EtOH treatment effects on locomotor activity and coordination in a heterogeneous population of mice

The experimental design was identical to that for experiment 2 (**Table 2**). A total of 36 WSC mice were tested, with a group size of 17–19 per treatment group. The average age of the mice at the initiation of testing was 87 ± 1 days, with a range of 78 to 96 days.

#### 2.4.4 Experiment 4: acute and repeated EtOH effects on locomotor activity and coordination in a heterogeneous population of mice given limited exposure to the grid test apparatus

The experimental design was similar to that for experiments 2 and 3, except that testing was limited to days 1–3 and 15 to examine whether the magnitude of sensitization/tolerance is impacted by exposure to the test apparatus (practice). The study schedule is summarized in **Table 3**. The CS group mice received saline on test days 1–3 and EtOH on test day 15. They were treated with saline in the colony room and returned to their home cages on days 4–14. The CE group mice received saline on test days 1 and 2 and EtOH on test days 3 and 15. They were treated with EtOH in the colony room and returned to their home cages on days 4–14. The test day data analyzed were for the first 10 min after treatment. Days 1 and 2 provided habituation and baseline activity data, respectively; day 3 provided a measure of initial response to EtOH in the CE group; day 15 provided a measure from which sensitization/tolerance could be determined in the CE group; day 15 also provided a measure of initial EtOH response in the CS group and a between-groups measure of sensitization/tolerance. A total of 40 WSC mice were tested; however, due to equipment malfunction, data from five mice were lost. Therefore, data from 35 mice were included in the final analysis, resulting in a group size of 19 for the CS group and 16 for the CE group. The average age of the mice at the initiation of testing was 86 ± 1 days, with a range of 76 to 91 days.

**Table 3:** Protocol for testing acute and repeated EtOH effects on locomotor activity and coordination in WSC mice with limited exposure to the test apparatus.

	Days				
	1	2	3	4–14	15
CS group					
**Injection**	SAL	SAL	SAL	SAL	EtOH
**Test?**	Y	Y	Y	N	Y
CE group					
**Injection**	SAL	SAL	EtOH	EtOH	EtOH
**Test?**	Y	Y	Y	N	Y

Both treatment groups were tested immediately after IP saline injection on test days 1 and 2. CS group mice were tested after IP saline injection on test day 3 and received home cage IP injections of saline on days 4–14, while the CE group mice were tested after IP injection of 2.5 g/kg EtOH on test day 3 and received home cage administration of 2.5 g/kg EtOH on days 4–14. On day 15, all mice received 2.5 g/kg EtOH immediately prior to testing.

SAL, saline; EtOH, ethanol; Y, mice were tested; N, mice were not tested.

### 2.5 Data analysis

Group sizes were based on expected error variance and treatment effect sizes from previous experiments. The primary statistical test was analysis of variance (ANOVA), and the statistical software was Statistica 13 (TIBCO Software, Palo Alto, CA, USA). For experiment 1, data were partitioned by treatment group (CS and CE) and strain. Raw data were directly compared or were compared using difference scores as indices of strain-dependent sensitivity. These measures are fully described in “Results”. For experiments 2–4, each of which involved only a single genotype and examined individual differences, day was included as a repeated measure. Significant two-way interactions were examined using simple main-effects analysis. Significant mean differences were detected by Newman–Keuls *post hoc* test. Difference scores were used with Pearson’s *r* statistic to calculate genetic correlations between traits from strain means or phenotypic correlations between traits from individual data points. The criterion for significance was set at *p* ≤ 0.05.

## 3 Results

### 3.1 Experiment 1: acute and repeated EtOH effects on locomotor activity and coordination in 15 inbred mouse strains

Data across days for each strain are plotted in **Supplementary Figures S1**–**S3**. These figures illustrate different patterns and magnitudes of response to EtOH vs. saline over days for locomotor activity counts (**Supplementary Figure S1**), number of errors (**Supplementary Figure S2**), and ataxia ratio (**Supplementary Figure S3**). To control for baseline locomotor activity differences and simplify strain comparisons, as in previous studies, we created difference scores for each mouse (EtOH score minus baseline) and then obtained strain means to index particular outcomes [see, e.g (21)]. Thus, an acute response to EtOH measure was derived by subtracting the day 2 saline baseline from the initial day 3 EtOH score for the CE group (day 3 - day 2) and by subtracting the day 2 saline baseline from the initial day 11 EtOH score for the CS group (day 11 - day 2). Magnitude of sensitization or tolerance with repeated EtOH administration was indexed by subtracting the initial day 3 EtOH score from the final day 11 EtOH score (day 11 - day 3) for locomotor activity or ataxia ratio in the CE group mice. Uncorrected day 11 locomotor data were compared between the CS and CE groups across strain as a between-groups measure of sensitization, and day 2 baseline data were statistically examined for group and strain differences.

#### 3.1.1 Locomotor activity counts

**Figure 1:**
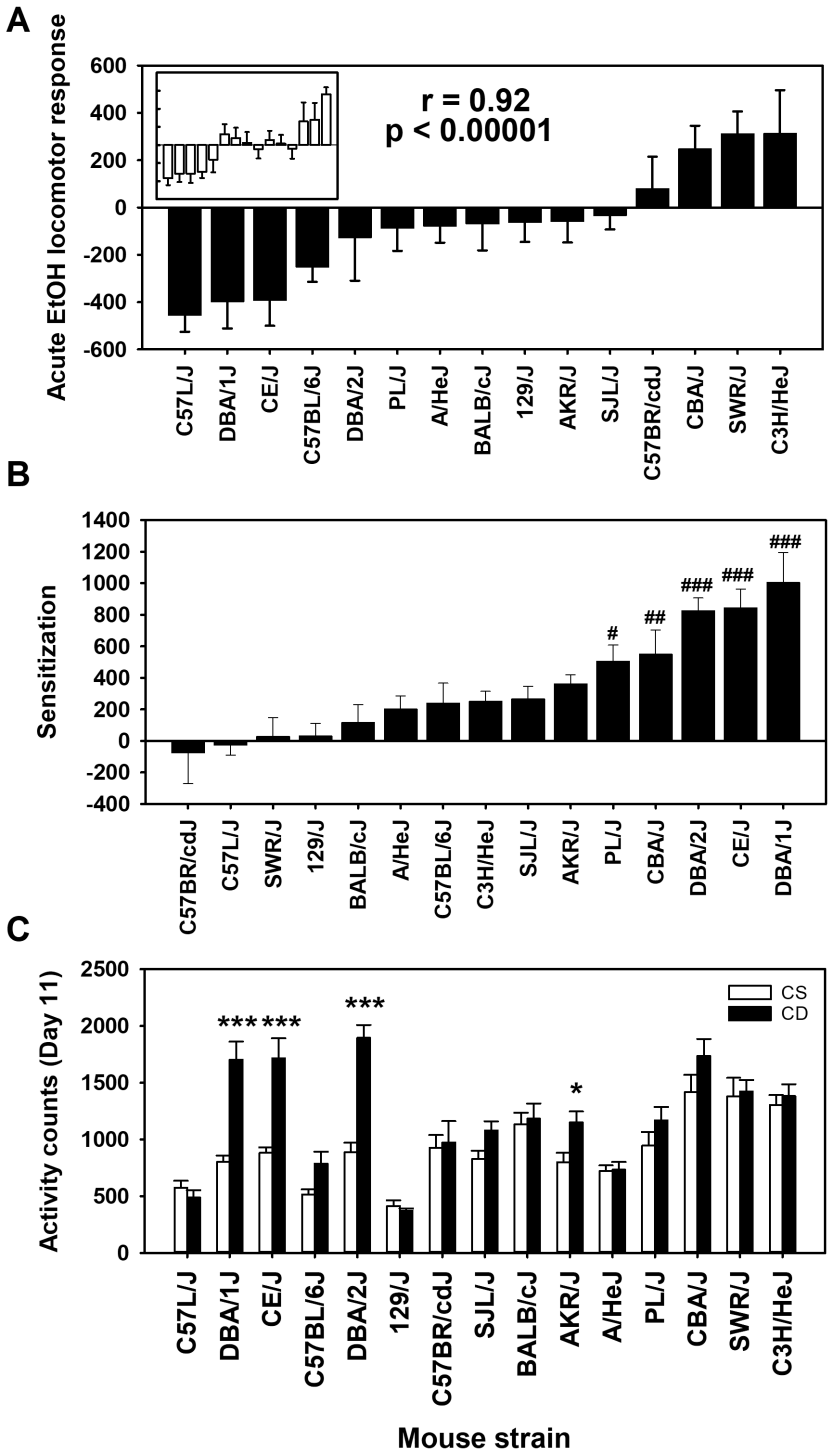
Mean EtOH-induced locomotor activity phenotypes in 15 inbred mouse strains. **(A)** Solid bars represent day 3 - day 2 acute EtOH locomotor response strain means for the chronic EtOH (CE) group. Open bars in the inset represent day 11 - day 2 acute EtOH locomotor response strain means for the chronic saline (CS) group. Strains are listed in the same order for the CS (inset) and CE group. The Pearson’s correlation (r) given is based on the strain means for the CE and CS groups. **(B)** Sensitization to EtOH measured as the change in locomotor activity response between the last and first EtOH administration (day 11 - day 3) in the CE group. **(C)** Locomotor activity on day 11 for CS and CE group mice. CS mice received EtOH for the first time on day 11, while CE group mice received EtOH for the fifth time on this day (see **Table 1** for treatment schedule). Shown are means ± SE. #*p* < 0.05, ##*p* < 0.01, ###*p* < 0.001 differences between that strain and the C57BR/cdJ strain (lowest amount of sensitization). *p < 0.05, ***p < 0.001 for the difference between the CS and CE groups within a given strain.

The mean acute EtOH locomotor activity responses for each strain and treatment group are shown in **Figure 1A**. There were significant differences in day 2 baseline activity level among the 15 inbred mouse strains (*F*_[14,261]_=10.85, *p* < 0.0001), justifying the need to correct for baseline differences to assess response to EtOH. Mean day 2 locomotor activity counts across the strains ranged from 403 ± 26 to 1,261 ± 83 (see **Supplementary Figure S1**); the correlation of CS and CE group strain means for day 2 was 0.96 (*p* < 0.00001). Positive acute EtOH response scores reflect locomotor stimulation, whereas negative scores reflect locomotor depression. An ANOVA identified a significant effect of strain (*F*_[14,261]_= 9.1, *p* < 0.0001). There was no significant effect of group, and there was no strain by group interaction, indicating that the acute EtOH locomotor responses were comparable for the CE (**Figure 1A**) and CS (**Figure 1A**, inset) groups. This is further reflected in the genetic correlation for the CE and CS strain distribution patterns for the acute EtOH response (*r* = 0.92; *p* < 0.00001).

Positive day 11 - day 3 scores in the CE group mice reflect sensitization to the locomotor stimulant effect of EtOH (**Figure 1B**). Higher activity counts on EtOH challenge day 11 in the CE group, compared to the CS group, provide additional evidence of sensitization (**Figure 1C**). For the CE group, there were significant strain differences (*F*_[14,132]_=8.4, *p* < 0.001), reflecting a range in magnitude of sensitization to EtOH. For comparison of the CE and CS groups on day 11, there was a significant strain × group interaction (*F*_[14,261]_=5.4, *p* < 0.001). Significantly more locomotor activity in the CE than in the CS group after EtOH challenge for four strains (**Figure 1C**) reflected significant locomotor sensitization.

#### 3.1.2 Grid test ataxia ratio

**Figure 2:**
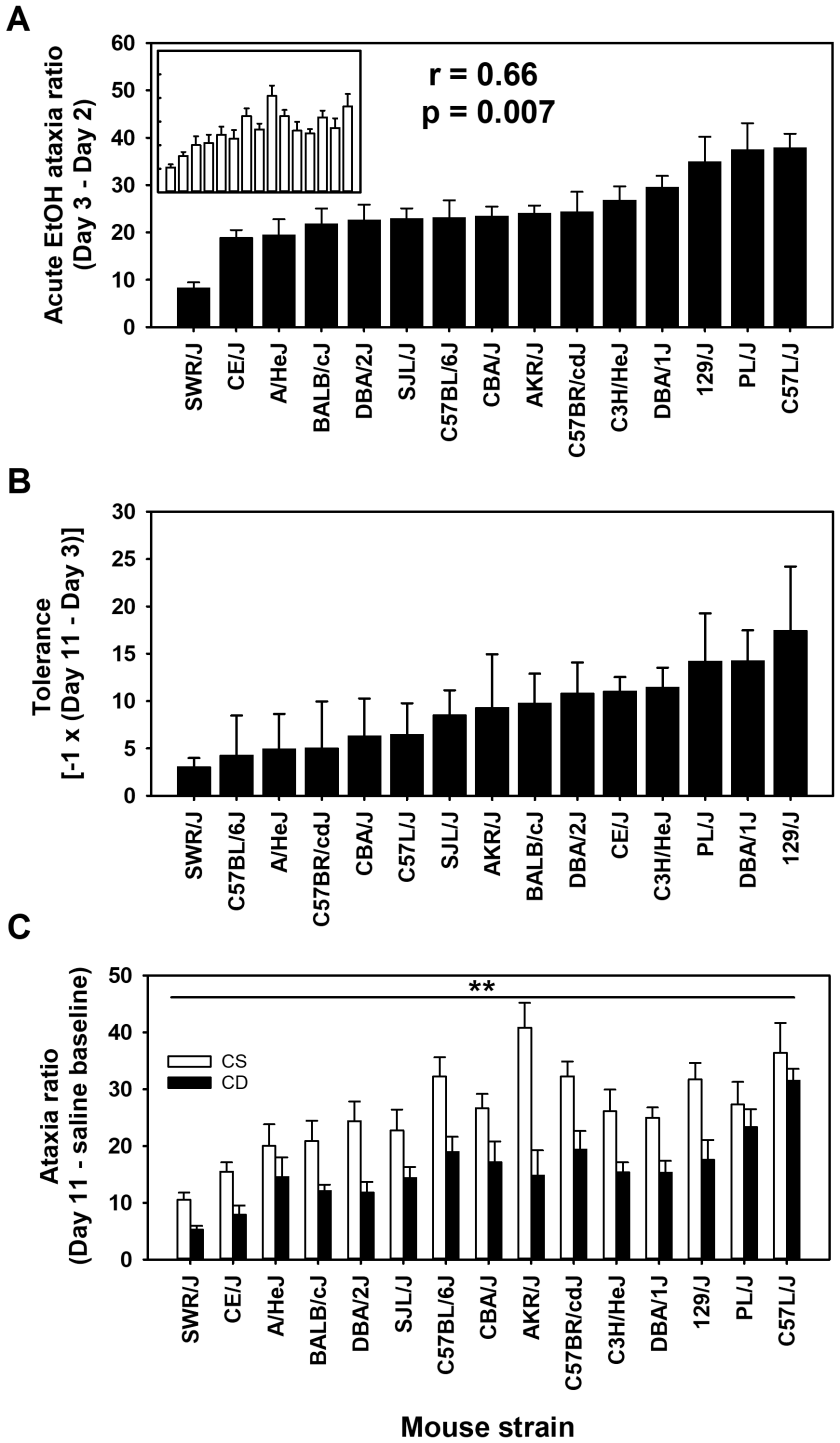
Mean EtOH-induced ataxia phenotypes in 15 inbred mouse strains. **(A)** Solid bars represent day 3 - day 2 acute EtOH ataxia ratio strain means for the chronic EtOH (CE) group. Open bars in the inset represent day 11 - day 2 acute EtOH ataxia ratio strain means for the chronic saline (CS) group. Strains are listed in the same order for the CS (inset) and CE group. Pearson’s correlation (*r*) given is based on the strain means for the CE and CS groups. **(B)** Tolerance to EtOH was measured as the change in the ataxia ratio between the last and first EtOH administration (day 11 - day 3) in the CE group. **(C)** Ataxia ratio on day 11 for CS and CE group mice. CS mice received EtOH for the first time on day 11, whereas CE group mice received EtOH for the fifth time on this day (see **Table 1** for treatment schedule). Shown are means ± SE. **p < 0.01 for the difference between CS and CE groups (main effect).

The ratio of foot slip errors to activity counts provides a normalized measure of ataxia that corrects for differences in locomotor activity because a higher level of locomotor activity provides a greater opportunity for foot slip errors to occur. The ataxia ratio can be thought of as missteps to steps taken. The uncorrected foot slip error strain and group means are shown in **Supplementary Figure S4**. For day 2 baseline ataxia ratio, there was a significant effect of strain (*F*_[14,261]_ = 14.5, *p* < 0.001), with means ranging from 0.26 ± 0.08 to 3.25 ± 0.50 (see **Supplementary Figure S3**); the correlation of CS and CE group strain means for day 2 was 0.96 (*p* < 0.00001). The mean acute EtOH scores for ataxia ratio corrected for baseline ratio (day 3 - day 2 for CE; day 11 - day 2 for CS) are shown in **Figure 2A** by strain and group. Larger values reflect greater EtOH-induced ataxia. An ANOVA identified a significant effect of strain (*F*_[14,261]_ = 9.3, *p* < 0.001) and a significant strain × group interaction (*F*_[14,261]_ = 1.9, *p* < 0.05), but no main effect of group. Further investigation into this interaction, using simple main effect analysis, illustrated that the CS and CE groups significantly differed in only two of the 15 strains tested. Those strains were PL/J (*p* < 0.05) and AKR/J (*p* < 0.001). The CE group of the PL/J strain displayed a greater amount of acute EtOH-induced ataxia (37.5 ± 5.5) than the CS group (27.3 ± 4.0). However, the AKR/J strain exhibited an opposite group difference, with the CE group displaying less EtOH-induced ataxia (24.1 ± 1.6) than the CS group (40.8 ± 4.3). The strain distribution patterns for the CS and CE groups are shown in **Figure 2A**. The genetic correlation was significant: *r* = 0.66 (*p* < 0.01).

To improve clarity, for the mean data reflecting the change in EtOH response after repeated administration, we have reversed the sign of the difference score [-1 × (day 11 - day 3)] so that larger scores reflect greater tolerance (**Figure 2B**). Although there was a range of scores, the effect of strain was not statistically significant for the magnitude of tolerance (*p* = 0.13). Likewise, when ataxia ratio data were compared for the CS and CE groups on EtOH challenge day 11 (**Figure 2C**), there was evidence for tolerance, but not for differences among strains in magnitude of tolerance. Thus, the CE group had lower ataxia ratios on day 11 compared to the CS group (*F*_[1,261]_ = 85.8, *p* < 0.001), reflecting tolerance, and there was a significant main effect of strain (*F*_[14,261]_ = 10.2, *p* < 0.001), but the strain × group interaction was not significant.

#### 3.1.3 Blood EtOH concentration

During the processing of the blood EtOH concentration (BEC) samples, two were lost (both from the CS group: one C57L/J sample and one A/HeJ sample). There was a significant main effect of strain for BEC in the samples collected at the end of the day 11 behavioral test session (*F*_[14,259]_ = 13.2, *p* < 0.001) as well as a significant group difference (*F*_[1,259]_ = 4.3, *p* < 0.05), with the CE group having a lower average BEC than the CS group (see **Supplementary Figure S5**). However, the group difference was only 0.04 mg/mL (mean ± SE for CE = 2.44 ± 0.02 mg/mL and CS = 2.48 ± 0.02 mg/mL). There was no significant strain × group interaction for BEC.

#### 3.1.4 Genetic and phenotypic correlations

The genetic correlations between the locomotor and ataxia measures, based on strain means, are presented in **Table 4**. Baseline activity was not significantly genetically correlated with any of the other measures. Larger acute EtOH ataxia ratios predicted greater tolerance (*r* = 0.59, *p* < 0.05); however, acute locomotor response to EtOH did not predict the magnitude of sensitization.

**Table 4:** Genetic correlations between baseline and EtOH-induced locomotor activity and ataxia traits based on means from 15 standard inbred strains.

	2	3	4	5	6
Activity					
1 Baseline	-0.19	0.45	0.24	-0.41	-0.20
2 Acute	1.00	-0.35	-0.26	-0.41	-0.24
3 Sensitization		1.00	-0.42	-0.01	0.41
Ataxia ratio					
4 Baseline			1.00	0.28	-0.23
5 Acute				1.00	0.59*
6 Tolerance					1.00

Baseline, saline day 2; Acute, initial EtOH response of CE mice on day 3 corrected for day 2 baseline; Sensitization or Tolerance, final EtOH response of CE mice on day 11 corrected for day 3 initial EtOH response.

N = 15 for all correlations. *p < 0.05 (critical value is r = 0.56 for p = 0.05).

Phenotypic correlations, which reflect both genetic and non-genetic contributions to variance, were calculated using data from all CE individuals across the inbred strains and are presented in **Table 5**. These correlations were based on data from CE group mice only because these mice contributed both measures and contributed baseline data. The number of individuals was 147, with 145 degrees of freedom for detecting a significant correlation, compared to a strain number of 15 and 13 degrees of freedom for the genetic correlations. Although there was no evidence for common genetic influence on baseline activity level and acute EtOH locomotor response, 28% of the variance in the acute response was predicted by baseline activity level for all CE mice (*r* = - 0.53, *p* < 0.001). Higher baseline activity level was significantly associated with lower acute EtOH ataxia ratio (*r* = -0.21, *p* < 0.01), but the predictive value was only 4%. We did not obtain evidence for a genetic relationship between acute EtOH-induced locomotor response and magnitude of sensitization, but in the phenotypic analysis, higher levels of acute locomotor response to EtOH were significantly associated with lower levels of sensitization (*r* = -0.23, *p* < 0.01), with only 5% of the variance in sensitization explained by initial locomotor response. The acute locomotor and ataxic responses to EtOH were also modestly negatively associated (*r* = -0.23, *p* < 0.01), but the amount of acute EtOH-induced ataxia robustly predicted the magnitude of tolerance (0.67, *p* < 0.001); acute ataxic response predicted 45% of the variance in tolerance level. Finally, the individual magnitude of EtOH-induced locomotor sensitization predicted about 10% of the variance in grid test ataxia tolerance (*r* = 0.32, *p* < 0.001).

**Table 5:** Phenotypic correlations between baseline and EtOH-induced locomotor activity and ataxia traits for individuals from 15 standard inbred strains.

	2	3	4	5	6
Activity					
1 Baseline	-0.53***	0.16	0.01	-0.21**	-0.06
2 Acute	1.00	-0.23**	0.01	-0.23**	-0.13
3 Sensitization		1.00	-0.26**	0.10	0.32***
Ataxia ratio					
4 Baseline			1.00	0.05	-0.17*
5 Acute				1.00	0.67***
6 Tolerance					1.00

Baseline, saline day 2; Acute, initial EtOH response of CE group mice on day 3 corrected for day 2 baseline; Sensitization or Tolerance, final EtOH response of CE group mice on day 11 corrected for day 3 initial EtOH response.

N = 147 for all correlations. *p < 0.05, **p < 0.01, ***p < 0.001 (critical value is r = 0.17 for p = 0.05).

### 3.2 Experiment 2: acute and repeated EtOH effects on locomotor activity and coordination in DBA/2J mice

In addition to the results described here for DBA/2J mice using a more robust procedure for inducing sensitization in this strain, see **Supplementary Figure S6** for the independent analysis of data from the DBA/2J mice from experiment 1 for comparison.

#### 3.2.1 Locomotor activity counts

**Figure 3:**
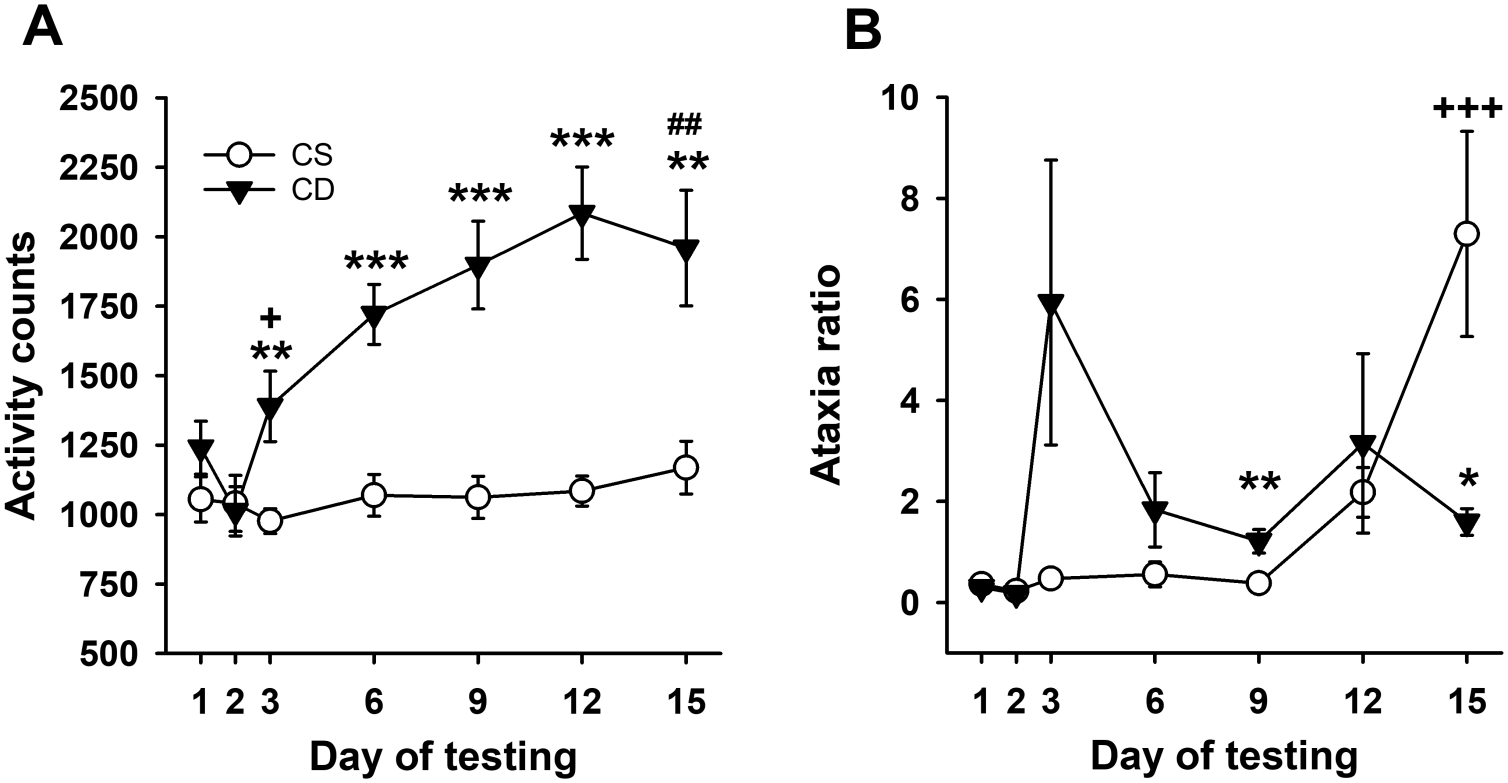
Mean EtOH-induced locomotor activity and ataxia phenotypes for DBA/2J mice. **(A)** Mean locomotor activity counts in CS and CE groups across days. **(B)** Mean ataxia ratio in CS and CE groups across days (see **Table 2** for treatment schedule). Shown are means ± SE. *p < 0.05, **p < 0.01, ***p < 0.001 significant difference between CE and CS groups for that test day; +*p* < 0.05, +++*p* < 0.001 significant acute response to EtOH (CS group day 15 vs. day 2; CE group day 3 vs. day 2); ##*p* < 0.01 significant sensitization in CE group (day 15 vs. day 3).

Statistical analyses reflected acute locomotor stimulation to EtOH in DBA/2J mice, with sensitization induced by repeated EtOH treatment. Repeated-measures ANOVA for locomotor activity counts across days (**Figure 3A**) identified a significant day × treatment group interaction (*F*_[6,84]_ = 8.4, *p* < 0.001). The CE and CS group mice had comparable activity levels on day 1 and day 2 (baseline). There was a significant group difference on day 3 (*p* = 0.008), reflecting a stimulant response to EtOH for the CE group compared to the locomotor activity level of the saline-treated CS group. A significant difference in activity level between the CS and CE groups was also present on days 6, 9, and 12. On day 15, when both groups were challenged with EtOH, the CE group exhibited more stimulation than the CS group (*p* = 0.004), demonstrating sensitization to the locomotor stimulant effect of EtOH in the CE group. Acute activation and sensitization were also confirmed by the results of repeated-measures ANOVA across days within the CE group, which identified a significant effect of day (*F*_[6,42]_ = 14.9, *p* < 0.001). Day 3 locomotor activity after EtOH was higher than baseline day 2 activity, and there was a pattern of increasing activity on each subsequent EtOH test day that leveled off on day 9. There was no significant effect of day in the repeated-measures ANOVA of the CS group data. The CS and CE groups did not significantly differ in the amount of acute EtOH-induced activation when their day 15 - day 2 and day 3 - day 2 scores were compared (128.75 ± 150.9 vs. 377.25 ± 119.9 for the CS and CE groups, respectively; *p* = 0.22).

#### 3.2.2 Grid test ataxia ratio

The pattern of change in EtOH-induced ataxia across days (**Figure 3B**) reflected rapid tolerance development. Uncorrected error count data are shown in **Supplementary Figure S7A**. Repeated-measures ANOVA for ataxia ratio (**Figure 3B**) identified a significant day × treatment group interaction (*F*_[6,84]_ = 4.4, *p* < 0.001). Ataxia ratios were comparable for the CS and CE groups on days 1 and 2. The CE group had a significantly larger ataxia ratio than the CS group on day 9, with a similar trend on day 3 (*p* = 0.07). The ataxia ratio for the CE group was smaller than for the CS group on EtOH challenge day 15, reflecting tolerance. Repeated-measures ANOVA for CE group data supported a strong statistical trend for changes across days (*F*_[6,42]_ = 2.2, *p* = 0.06). Based on this strong trend and our *a priori* interest in tolerance development, the day 3 and day 15 means were compared, and there was a trend for a significant difference (*p* = 0.11). For the CS group, there was a significant main effect of day (*F*_[6,42]_ = 10.3, *p* < 0.001), with a larger ataxia ratio on EtOH challenge day 15 than on any of the previous days. The ataxia ratios after acute EtOH administration were not significantly different for the CS (day 15 - day 2) and CE (day 3 - day 2) groups (7.06 ± 2.08 vs. 5.76 ± 2.84 for the CS and CE groups, respectively; *p* = 0.59).

#### 3.2.3 Blood EtOH concentration

The CE and CS groups differed in BECs from samples obtained at the end of EtOH challenge day 15 (*F*_[1,14]_ = 1083, *p* < 0.01; mean ± SE for CE = 2.10 ± 0.24 mg/mL and CS = 2.99 ± 0.14 mg/mL), suggesting that repeated EtOH exposure may have increased the rate of EtOH clearance.

### 3.3 Experiment 3: acute and repeated EtOH effects on locomotor activity and coordination in WSC mice

#### 3.3.1 Locomotor activity counts

**Figure 4:**
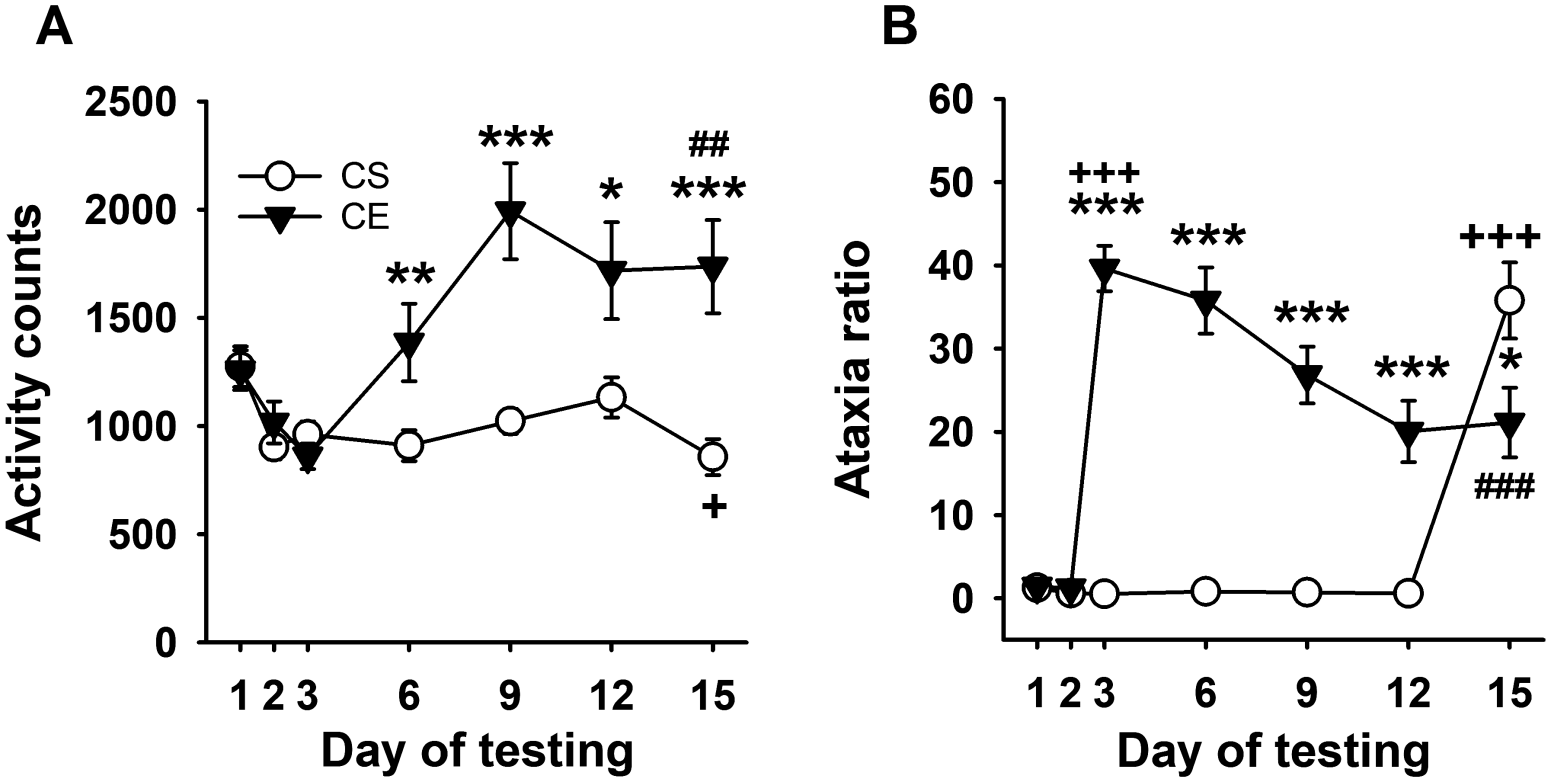
Mean EtOH-induced locomotor activity and ataxia phenotypes for WSC mice. **(A)** Mean locomotor activity counts in CS and CE groups across days. **(B)** Mean ataxia ratio in CS and CE groups across days (see **Table 2** for treatment schedule). Shown are means ± SE. *p < 0.05, **p < 0.01, ***p < 0.001 for the difference between CE and CS groups for that test day; +*p* < 0.05, +++*p* < 0.001 for the acute response to EtOH (CS group day 15 vs. day 2; CE group day 3 vs. day 2); ##*p* < 0.01, ###*p* < 0.001 for sensitization or tolerance in the CE group (day 15 vs. day 3).

WSC mice did not exhibit an acute stimulant response to initial EtOH treatment; however, a stimulant response to EtOH developed and increased across days (**Figure 4A**), reflecting sensitization. Repeated-measures ANOVA identified a significant day × treatment group interaction (*F*_[6,198]_ = 8.7, *p* < 0.001). There were no significant differences between the two groups on saline days 1 and 2 or on day 3 when CE group mice received EtOH. Significant differences in activity level were present between the CS and CE groups on days 6, 9, 12, and 15, with higher activity counts in CE mice compared to CS mice on all days. The larger EtOH response in CE mice compared to CS mice on day 15 reflected significant sensitization in the CE group. Repeated-measures ANOVA for data within each treatment group identified a significant effect of day in both the CE (*F*_[6,96]_ = 8.8, *p* < 0.001) and CS (*F*_[6,102]_ = 7.7, *p* < 0.001) groups. In the CE group, there was an increasing pattern of stimulation across days that peaked on day 9; the activity levels were significantly higher on EtOH treatment days 6–15 compared to the initial EtOH treatment on day 3, supporting sensitization. The CS group did not exhibit sensitivity to the acute locomotor stimulant effect of EtOH; in fact, their mean activity level was significantly reduced on EtOH challenge day 15 compared to saline baseline day 2.

#### 3.3.2 Grid test ataxia ratio

Uncorrected error count data are shown in **Supplementary Figure S7A**. For ataxia ratio (**Figure 4B**), decreasing ataxia mirrored increasing sensitization (**Figure 4A**). Repeated-measures ANOVA identified a significant day × treatment group interaction (*F*_[6,198]_ = 36.9, *p* < 0.001). There was no significant group difference in mean ataxia ratio on day 1 or 2, but the groups differed on subsequent days 3-12, with greater ataxia in the CE than the CS group. The lower ataxia ratio in the CE group compared to the CS group on EtOH challenge day 15 supported tolerance to the ataxic effect of EtOH in the CE group. Repeated-measures ANOVA supported changes across day for the CE group (*F*_[6,96]_ = 27.5, *p* < 0.001) and CS group (*F*_[6,102]_ = 59.7, *p* < 0.001). CE group ataxia ratio was elevated above baseline on all EtOH treatment days but progressively decreased below their acute EtOH day 3 ataxia ratio. Significantly lower ataxia on day 15 than on acute day 3 supported the development of tolerance within the CE group. The ataxia ratio of the CS group on EtOH challenge day 15 was significantly greater than on any of the saline treatment days, confirming acute EtOH-induced ataxia.

#### 3.3.3 Blood EtOH concentrations

There was no significant group difference in BECs obtained from samples taken after testing on day 15 (mean ± SE for CE = 1.61 ± 0.13 mg/mL and CS = 1.67 ± 0.10 mg/mL; *p* = 0.70); thus, EtOH clearance was not significantly impacted by repeated EtOH exposure in the WSC mice.

#### 3.3.4 Phenotypic correlations

Similar to experiment 1, potential relationships between the various measures were examined for data from individuals within the CE group and provided some support for the hypothesis that locomotor sensitization corresponds with tolerance development to the ataxic effects of EtOH. Correlations are given in **Table 6**. As in experiment 1, higher levels of baseline activity predicted lower locomotor responses to acute EtOH (*r* = -0.87, *p* < 0.001), with baseline activity explaining 76% of the variance in EtOH response for the *n* = 16 WSC mice. No other traits were significantly correlated with baseline activity. Magnitude of sensitization predicted 44% of the variance in magnitude of tolerance (*r* = 0.66, *p* < 0.01) and 38% of the variance in acute ataxic response to EtOH (*r* = 0.62, *p* < 0.01). Finally, acute EtOH ataxia ratio predicted 50% of the variance in magnitude of tolerance (*r* = 0.71, *p* < 0.01).

**Table 6:** Phenotypic correlations between baseline and EtOH-induced locomotor activity and ataxia traits for individual WSC mice.

	2	3	4	5	6
Activity					
1 Baseline	-.87***	0.42	-0.14	-0.11	0.24
2 Acute	1.00	-0.28	-0.05	0.16	-0.07
3 Sensitization		1.00	-0.14	0.62**	0.66**
Ataxia ratio					
5 Baseline			1.00	0.22	0.18
5 Acute				1.00	0.71**
6 Tolerance					1.00

Baseline, saline day 2; Acute, initial EtOH response of CE group mice on day 3 corrected for day 2 baseline; Sensitization or Tolerance, final EtOH response of CE group mice on day 11 corrected for day 3 initial EtOH response.

N = 16 for all correlations. **p < 0.01, ***p < 0.001 (critical value is r = 0.50 for p = 0.05).

### 3.4 Experiment 4: acute and repeated EtOH effects on locomotor activity and coordination in WSC mice with limited exposure to the grid test apparatus

#### 3.4.1 Locomotor activity counts

**Figure 5:**
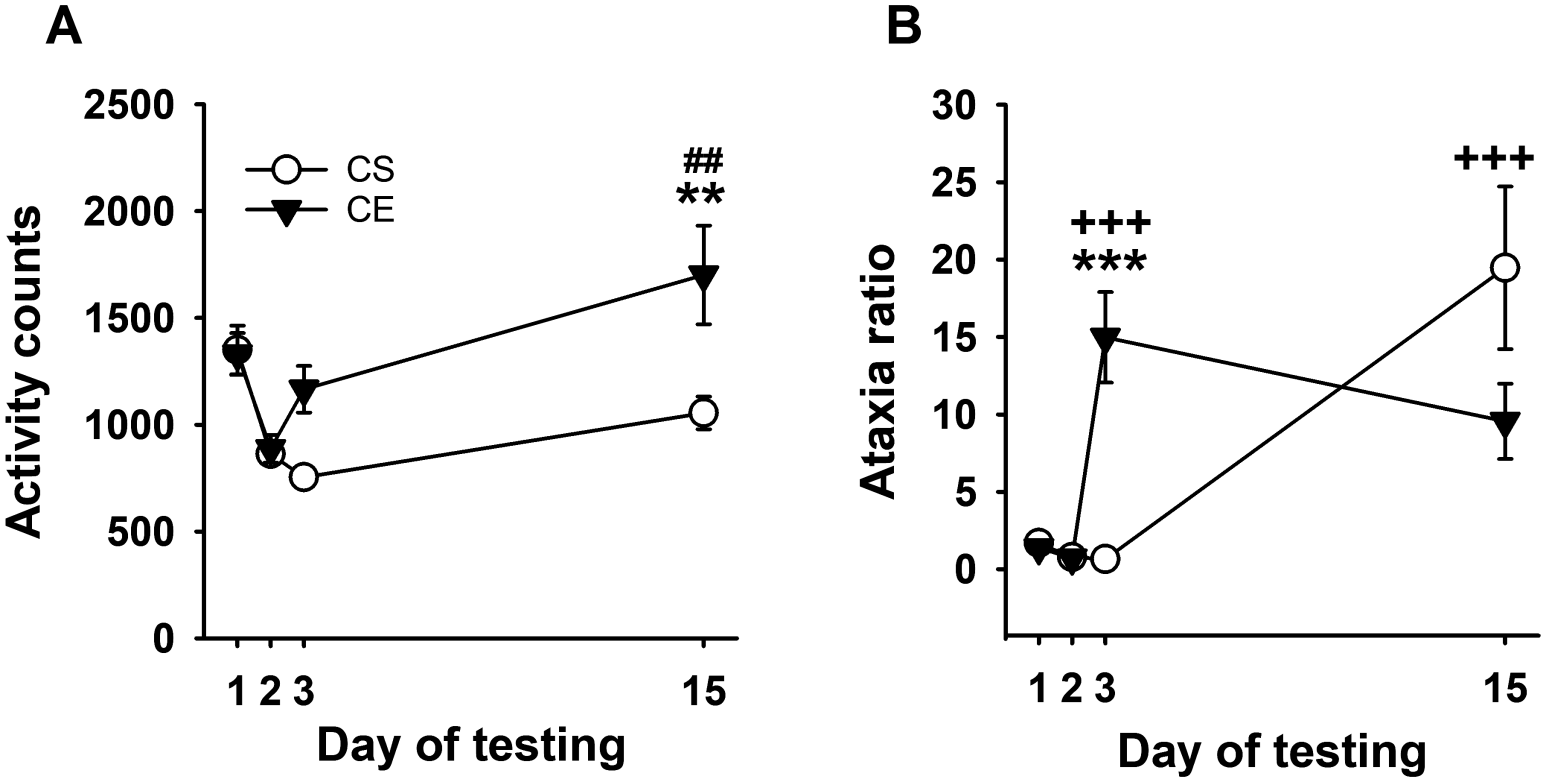
Mean EtOH-induced locomotor activity and ataxia phenotypes for WSC mice in a limited test apparatus exposure procedure. **(A)** Mean locomotor activity in CS and CE groups across days. **(B)** Mean ataxia ratio in CS and CE groups across days (see **Table 3** for treatment schedule). Shown are means ± SE. **p < 0.01, ***p < 0.001 for difference between CE and CS groups on that test day; +++*p* < 0.001 for the acute response to EtOH (CS group day 15 vs. day 2; CE group day 3 vs. day 2); ##*p* < 0.01 for sensitization in the CE group (day 15 vs. day 3).

Repeated-measures ANOVA identified a significant day × treatment group interaction (*F*_[3,102]_ = 6.7, *p* < 0.001) for activity counts (**Figure 5A**). Similar to Experiment 3, the CS and CE group WSC mice had similar levels of activity on baseline days 1 and 2, and the CE group did not exhibit a significant stimulant response to acute EtOH on day 3 compared to the activity level of the saline-treated CS group. Repeated EtOH treatment of CE group mice, without repeated testing, resulted in sensitization to the stimulant effect of EtOH (day 15 compared to day 3). Between-groups sensitization was also present on EtOH challenge day 15. Repeated-measures ANOVA detected a significant main effect of day for both the CE (*F*_[3,51]_ = 7.0, *p* < 0.001) and CS (*F*_[3,51]_ = 14.0, *p* < 0.001) groups. The significantly larger mean activity level on day 15 than on day 3 supported sensitization in the CE group. For CS mice, there was no significant difference between activity levels on days 15 and 2, indicating that, similar to the CE group, EtOH did not have an acute stimulant effect in the CS group.

#### 3.4.2 Grid test ataxia ratio

Uncorrected error count data are shown in **Supplementary Figure S7C**. The results for the ataxia ratio supported the development of tolerance (**Figure 5B**). Repeated-measures ANOVA identified a significant day × treatment group interaction (*F*_[3,90]_=7.2, *p* < 0.001), with a larger ataxia ratio in CE than CS group mice on day 3, demonstrating an acute ataxic effect of EtOH in the CE group. Repeated-measures ANOVA identified a main effect of day for both the CE (*F*_[3,42]_ = 11.8, *p* < 0.001) and CS (*F*_[3,48]_ = 10.2, *p* < 0.001) groups. For the CE group, there was a significant increase in ataxia after acute EtOH on day 3 compared to day 2. Although there was a negative slope, there was no significant difference between day 3 and day 15 ataxia ratio, suggesting that repeated testing may be needed for robust tolerance development. For CS group data, there was a larger ataxia ratio on day 15 after acute EtOH treatment compared to day 2 baseline.

#### 3.4.3 Blood ethanol concentration

There was no significant group difference in BECs obtained from samples taken after testing on day 15 (mean ± SE for CE = 2.21 ± 0.14 mg/mL and CS = 2.35 ± 0.13 mg/mL); thus, EtOH clearance was not significantly impacted by repeated EtOH exposure in the WSC mice.

#### 3.4.4 Phenotypic correlations

Phenotypic correlations for the CE group data are given in **Table 7**. There were two significant correlations for this final group of 16 WSC mice (three were dropped due to incomplete data for correlations). Baseline ataxia ratio predicted 40% of the variance in acute ataxic response to EtOH (*r* = 0.63, *p* < 0.01), and acute ataxic response to EtOH predicted 40% of the variance in magnitude of tolerance (*r* = -0.63, *p* < 0.01). Using this protocol in which mice were tested on only two EtOH treatment days, there was no phenotypic relationship found between the magnitude of EtOH-induced sensitization and tolerance.

**Table 7:** Phenotypic correlations between baseline and EtOH-induced locomotor activity and ataxia traits for individual WSC mice with limited exposure to the test apparatus.

	2	3	4	5	6
Activity					
1 Baseline	0.05	-0.37	-0.16	-0.13	-0.29
2 Acute	1.00	-0.11	0.22	0.07	0.07
3 Sensitization		1.00	-0.05	0.32	-0.19
Ataxia ratio					
4 Baseline			1.00	0.63**	-0.43
5 Acute				1.00	-0.63**
6 Tolerance					1.00

Baseline, saline day 2; Acute, initial EtOH response of CE group mice on day 3 corrected for day 2 baseline; Sensitization or Tolerance, final EtOH response of CE group mice on day 11 corrected for day 3 initial EtOH response.

N = 16 for all correlations. **p < 0.01 (critical value is r = 0.50 for p = 0.05).

## 4 Discussion

Tolerance to EtOH has been included in the Diagnostic and Statistical Manual of Mental Disorders as a key criterion for the diagnosis of alcohol use disorder (AUD) for decades (57). Sensitization is thought to contribute to the maintenance of EtOH seeking, increases in the rewarding effects of EtOH, and has been associated with both future heavy drinking and AUD (2, 58), but little research has investigated the relationship between these two processes in either humans or animal models. The goal of the current set of studies was to investigate the hypothesis that the magnitude of behavioral sensitization to EtOH is related to the magnitude of behavioral tolerance to EtOH. If the two phenotypes change in concert with each other, this would suggest that locomotor sensitization occurs as the sedative–ataxic effects of EtOH wane. On the other hand, lack of coordination would suggest independent underlying mechanisms. To examine this hypothesis, sensitization and tolerance were simultaneously measured using a grid test apparatus capable of providing data on locomotor activity and coordination. Using this unique approach, associations between the two phenotypes were studied in a genetically diverse panel of 15 inbred mouse strains, in a strain with high susceptibility to both traits (19, 29–38), and in a genetically heterogeneous stock (39). We hypothesized that increasing amounts of sensitization would mirror increasing amounts of tolerance. We found mixed evidence for an association.

### 4.1 Genetic relationships

Data from 15 inbred mouse strains were initially examined using strain means to calculate genetic correlations. The correlation between the sensitization and tolerance measures was in the hypothesized direction with greater sensitization associated with greater tolerance; however, with a group size of 15 strains, the genetic correlation of 0.41 (*p* = 0.13) was not statistically significant. Furthermore, although there was a range of means for both phenotypes, there was a significant effect of strain for sensitization, but not tolerance. A significant genetic correlation might be found in a study with a larger number of strains and a larger group size to reduce error variation, particularly for the tolerance measure. Some of that variation could be associated with biological sex. Our study was not adequately powered to study sex differences, but they are something to consider as a source of variation in future studies.

Other traits examined for potential relationships with each other and with sensitization and tolerance were levels of baseline activity and ataxia and the acute effects of EtOH on these measures. For some measures, reliability of strain means could be examined by comparing CS and CE groups—for example, the genetic correlation, based on strain means, for day 2 baseline activity level for CS and CE group mice was 0.96 (*p* < 0.00001). In addition, although the acute EtOH response of CE and CS group mice was measured at different stages of the study, on day 3 in CE and day 11 in CS, the genetic correlation was highly significant (*r* = 0.92, *p* < 0.00001). The genetic correlation for the two groups was also significant for acute EtOH-induced ataxia (*r* = 0.66, *p* < 0.01). Across measures, the only significant genetic correlation was between the amount of ataxia displayed upon initial exposure to EtOH and the amount of tolerance development, such that greater acute ataxia was associated with greater tolerance, predicting about 35% of the variance. A possible explanation for this relationship is that higher initial values allow for a greater change to lower values. However, no such relationship was found for acute locomotor response and sensitization. Thus, it was not systematically the case that strains with lower acute EtOH locomotor scores had higher sensitization scores.

### 4.2 Phenotypic relationships between EtOH-induced sensitization and tolerance

The data from the 15 standard inbred strains were next examined without consideration of strain, allowing us to determine phenotypic correlations for a large genetically diverse population based on values for each individual mouse. In this case, there was a significant positive correlation between magnitude of sensitization and tolerance, with the magnitude of tolerance explaining about 10% of the variance in sensitization. In addition, within the CE group mice, baseline activity level predicted 28% of the variance in acute activity response to EtOH (*r* = -0.53, *p* < 0.001). This relationship was negative so that a higher baseline was associated with a lower stimulant response, indicating a potential ceiling effect. Mice with high levels of baseline activity may be limited in the amount of increase that is physically possible. Acute EtOH-induced ataxia ratio was also inversely related to baseline activity level (i.e., lower day 3 – day 2 ataxia ratio score was associated with higher day 2 baseline activity; *r* = -0.21, *p* < 0.01). This relationship derives from mice with higher activity levels committing more foot slips even under saline treatment conditions and supports the need to correct the data for differences in activity. Thus, for mice with greater day 2 baseline activity levels, a larger day 2 baseline ataxia ratio is subtracted from the day 3 EtOH ataxia ratio. However, the relationship is not particularly strong with baseline activity differences explaining only 5% of the variance in acute EtOH-induced ataxia ratio. Finally, similar to the correlation found for strain means, ataxia upon initial exposure to EtOH predicted about 45% of the variance in tolerance.

For experiment 2, data were collected in the DBA/2J strain, known to develop robust EtOH-induced sensitization (21, 29, 32, 34, 52). The 2.5-g/kg dose of EtOH used in this study induced a significant acute stimulant response, and daily administration resulted in robust sensitization that peaked on day 12 of testing. The CS and CE group mice exhibited a similar acute stimulant response to EtOH, reflecting the reliability of this effect in different groups of DBA/2J mice. There was a strong acute ataxic response to EtOH, and the difference between the acute response of the CS group and the final response of the CE group reflected significant tolerance. A strong statistical trend for tolerance development was found across repeated administration in the CE group (*p* = 0.06), but there was considerable variability in response (see **Figure 3**). The patterns of response across days for sensitization and tolerance were somewhat different, with sensitization continuing to increase across days 6–12, but maximum tolerance appeared to occur on day 6.

The heterogeneous stock represents a genetically diverse population, but unlike the standard inbred strains, the mice are not homozygous at all loci and, in that respect, are more like a human population. The advantages of using a genetically heterogeneous mouse population have been discussed (59–61). The WSC population used for our study was derived from eight inbred strains, six of which are closely related to the inbred strains included in experiment 1 (A, AKR, BALB/c, C3H/2, C57BL, and DBA/2); however, suppliers have changed across the years since the WSC were developed, and there is likely to have been some genetic drift. Two strains (IS/Bi and RIII) no longer exist and contributed unique genetic material compared to the panel of inbred strains that we tested. Although WSC mice did not exhibit mean acute stimulation to EtOH, they did exhibit locomotor sensitization. These mice exhibited a robust ataxic response to EtOH and gradual tolerance. When we examined the phenotypic relationship between EtOH-induced sensitization and tolerance, there was a significant positive correlation, though the patterns across time did not completely correspond; thus, maximum sensitization occurred on day 9, whereas maximum tolerance occurred on day 12. It is possible that floor or ceiling effects could impact these patterns, even if some of the same underlying neurobiological mechanisms are involved, contributing to the day on which the maximum level of a trait is observed.

Several of the significant phenotypic correlations between traits found for the inbred strains were also found for the WSC. Thus, there was an inverse relationship between baseline activity and acute locomotor response to EtOH, and acute ataxia was associated with magnitude of tolerance. Although magnitude of sensitization and tolerance were significantly correlated in both studies, sensitization explained about 10% of the variance in EtOH-induced tolerance in the inbred strains, whereas it explained 44% of the variance in the WSC. There were also some differences across the two studies (see **Tables 5**, **6**)—for example, an association found in the WSC that was not found in the inbred strains was a positive correlation between magnitude of sensitization and the acute EtOH ataxia ratio. Since higher acute ataxia is also related to greater tolerance, this may be associated with the stronger correspondence between sensitization and tolerance in the WSC study. Genetic and non-genetic factors could contribute to these several differences since these are phenotypic, rather than genetic, correlations.

### 4.3 Behavioral practice and the development of EtOH-induced sensitization and tolerance

One question that we posed is whether behavioral practice or number of exposures to the test apparatus under EtOH treatment might impact the relationship between sensitization and tolerance. In experiment 4, we limited exposure to the apparatus while under the influence of EtOH by giving several injections in the home cage. The mice in this study still developed both within-group and between-group locomotor sensitization and tolerance. However, although the correlation between acute EtOH-induced ataxia and ataxia tolerance remained in this study, no significant correlation was found between magnitude of EtOH-induced sensitization and tolerance. This suggests a role for exposure to the test apparatus during EtOH exposure in the relationship between these two traits. However, it is notable that the acute ataxic response of this set of WSC mice was attenuated by more than 50% compared to the mice in experiment 3, limiting the amount of tolerance development possible.

### 4.4 Conclusions

The current set of studies utilized multiple mouse populations to simultaneously measure EtOH-induced sensitization and tolerance and determine whether there was coordinated development of the two traits across repeated EtOH treatments. The majority of consideration of the relationship between these two phenotypes in the literature has been for psychostimulants and opiates (62–64). Several factors have been suggested to contribute to the development of tolerance and sensitization, including changes in the pharmacokinetics and pharmacodynamics of the drug as well as conditioning and state-dependent learning (practice). Whether they play common roles in the two phenotypes is not known. A source of common influence is shared genetic factors that impact both traits. Based on the data presented in this paper and consistent with our prior study in RI strains (21), it appears that there are independent genetic factors that impact the magnitude of EtOH-induced sensitization and tolerance. Therefore, both sensitization and tolerance need to be considered as independent processes that could impact vulnerability to addiction. However, our data support the conclusion that individual differences, which could be due to genetic or environmental influences (or both), impact the relationship between magnitude of sensitization and tolerance. This relationship was disrupted when EtOH treatment was dissociated from the test environment, suggesting that drug–environment interactions are important. Lastly, we found some evidence for effects of repeated EtOH administration on final BEC, such that BECs were lower in the CE group compared to the CS group mice for both the inbred strain panel and the DBA/2J studies, but not the studies in WSC mice. Therefore, the finding was inconsistent in the presence of significant sensitization and tolerance across the studies.

There has been considerable research conducted on mechanisms underlying EtOH-induced sensitization and tolerance, both of which have been the subject of reviews (51, 57, 65). Although there are some common findings for neurotransmitters, membrane-bound channels, and other mechanisms that impact the two traits, it is not clear that they are two features of the same process. Our experiments were not designed to address the molecular bases of these traits. Rather, our behavioral approach of simultaneously measuring these traits sheds light on this question in a unique way. There were some methodological variations across the studies performed that could have impacted our results—for example, there were differences in EtOH dose and frequency of treatment across studies. In each study, significant sensitization and tolerance were found, allowing for the examination of the hypothesis that the two traits reflect a common process. We obtained some data to support a genetic or phenotypic relationship between the two traits. Other data showed different timeframes of sensitization and tolerance development, indicating that these effects of repeated EtOH exposure are not perfectly coordinated and appear to involve at least some different mechanisms. An alternative interpretation is that floor or ceiling effects impact these patterns, contributing to the day on which the maximum level of a trait was observed. One limitation of these studies is that they were conducted only in male mice, leaving the question of generality across sexes unanswered. Our previous similar study in C57BL/6J by DBA/2J recombinant inbred strains tested female mice and did not obtain evidence supporting the hypothesis that sensitization and tolerance develop in concert (21). The potential sources of differences in results across these studies are sex and genetics since the current study included more genetically diverse populations of mice.

## Data Availability

The raw data supporting the conclusions of this article will be made available by the authors, without undue reservation.

## References

[1] Allen HC Weafer J Wesley MJ Fillmore MT. Heightened motor impairment as a protective factor against heavy drinking in individuals with high alcohol-induced disinhibition. Alcohol Clin Exp Res (Hoboken). (2023) 47:414–24. doi: 10.1111/acer.15003 PMC9991985 36549890

[2] King AC McNamara PJ Hasin DS Cao D. Alcohol challenge responses predict future alcohol use disorder symptoms: a 6-year prospective study. Biol Psychiatry. (2014) 75:798–806. doi: 10.1016/j.biopsych.2013.08.001 24094754 PMC4280017

[3] King AC Hasin D O'Connor SJ McNamara PJ Cao D. A prospective 5-year re-examination of alcohol response in heavy drinkers progressing in alcohol use disorder. Biol Psychiatry. (2016) 79:489–98. doi: 10.1016/j.biopsych.2015.05.007 PMC4644521 26117308

[4] Quinn PD Fromme K. Subjective response to alcohol challenge: a quantitative review. Alcohol Clin Exp Res. (2011) 35:1759–70. doi: 10.1111/acer.2011.35.issue-10 PMC3183255 21777258

[5] Schuckit MA Smith TL Kalmijn J Trim RS Cesario E Saunders G. Comparison across two generations of prospective models of how the low level of response to alcohol affects alcohol outcomes. J Stud Alcohol Drugs. (2012) 73:195–204. doi: 10.15288/jsad.2012.73.195 22333327 PMC3281979

[6] Schuckit MA Smith TL Clarke D Mendoza LA Kawamura M Schoen L. Predictors of increases in alcohol problems and alcohol use disorders in offspring in the San Diego prospective study. Alcohol Clin Exp Res. (2019) 43:2232–41. doi: 10.1111/acer.14164 PMC6779494 31454095

[7] Belknap JK. The grid test: A measure of alcohol- and barbiturate-induced behavioral impairment in mice. Behav Res Methods Instruments. (1975) 7:66–7. doi: 10.3758/BF03201299

[8] Dudek BC Phillips TJ. Distinctions among sedative, disinhibitory, and ataxic properties of ethanol in inbred and selectively bred mice. Psychopharmacol (Berl). (1990) 101:93–9. doi: 10.1007/BF02253724 2343078

[9] Funada M Takebayashi-Ohsawa M Tomiyama KI. Synthetic cannabinoids enhanced ethanol-induced motor impairments through reduction of central glutamate neurotransmission. Toxicol Appl Pharmacol. (2020) 408:115283. doi: 10.1016/j.taap.2020.115283 33068620

[10] Krahe TE Filgueiras CC da Silva Quaresma R Schibuola HG Abreu-Villaça Y Manhães AC. Energy drink enhances the behavioral effects of alcohol in adolescent mice. Neurosci Lett. (2017) 651:102–8. doi: 10.1016/j.neulet.2017.04.050 28456714

[11] Masur J Boerngen R. The excitatory component of ethanol in mice: a chronic study. Pharmacol Biochem Behav. (1980) 13:777–80. doi: 10.1016/0091-3057(80)90206-3 7208545

[12] McClearn GE Anderson SM. Genetics and ethanol tolerance. Drug Alcohol Depend. (1979) 4:61–76. doi: 10.1016/0376-8716(79)90041-3 574446

[13] Phillips TJ Burkhart-Kasch S Terdal ES Crabbe JC. Response to selection for ethanol-induced locomotor activation: genetic analyses and selection response characterization. Psychopharmacol (Berl). (1991) 103:557–66. doi: 10.1007/BF02244259 2062990

[14] Didier N Vena A Feather AR Grant JE King AC. Holding your liquor: Comparison of alcohol-induced psychomotor impairment in drinkers with and without alcohol use disorder. Alcohol Clin Exp Res (Hoboken). (2023) 47:1156–66. doi: 10.1111/acer.15080 37330919

[15] Ferreira S Soares LM Lira CR Yokoyama TS Engi SA Cruz FC. Ethanol-induced locomotor sensitization: Neuronal activation in the nucleus accumbens and medial prefrontal cortex. Neurosci Lett. (2021) 749:135745. doi: 10.1016/j.neulet.2021.135745 33610663

[16] Linsenbardt DN Boehm SL 2nd. Determining the heritability of ethanol-induced locomotor sensitization in mice using short-term behavioral selection. Psychopharmacol (Berl). (2013) 230:267–78. doi: 10.1007/s00213-013-3151-4 PMC3809338 23732838

[17] Masur J Oliveira de Souza ML Zwicker AP. The excitatory effect of ethanol: absence in rats, no tolerance and increased sensitivity in mice. Pharmacol Biochem Behav. (1986) 24:1225–8. doi: 10.1016/0091-3057(86)90175-9 3725828

[18] Pastor R Reed C Meyer PJ McKinnon C Ryabinin AE Phillips TJ. Role of corticotropin-releasing factor and corticosterone in behavioral sensitization to ethanol. J Pharmacol Exp Ther. (2012) 341:455–63. doi: 10.1124/jpet.111.190595 PMC3336812 22333290

[19] Phillips TJ Dickinson S Burkhart-Kasch S. Behavioral sensitization to drug stimulant effects in C57BL/6J and DBA/2J inbred mice. Behav Neurosci. (1994) 108:789–803. doi: 10.1037/0735-7044.108.4.789 7986372

[20] Phillips TJ Huson M Gwiazdon C Burkhart-Kasch S Shen EH. Effects of acute and repeated ethanol exposures on the locomotor activity of BXD recombinant inbred mice. Alcohol Clin Exp Res. (1995) 19:269–78. doi: 10.1111/j.1530-0277.1995.tb01502.x 7625557

[21] Phillips TJ Lessov CN Harland RD Mitchell SR. Evaluation of potential genetic associations between ethanol tolerance and sensitization in BXD/Ty recombinant inbred mice. J Pharmacol Exp Ther. (1996) 277:613–23. 8627538

[22] van Ingelgom T Didone V Godefroid L Quertemont É. Effects of social housing conditions on ethanol-induced behavioral sensitization in Swiss mice. Psychopharmacol (Berl). (2024) 241:987–1000. doi: 10.1007/s00213-024-06527-7 38206359

[23] Gallaher EJ Gionet SE. Initial sensitivity and tolerance to ethanol in mice genetically selected for diazepam sensitivity. Alcohol Clin Exp Res. (1988) 12:77–80. doi: 10.1111/j.1530-0277.1988.tb00136.x 2831751

[24] Kreifeldt M Cates-Gatto C Roberts AJ Contet C. BK channel β1 subunit contributes to behavioral adaptations elicited by chronic intermittent ethanol exposure. Alcohol Clin Exp Res. (2015) 39:2394–402. doi: 10.1111/acer.12911 PMC4712106 26578345

[25] Le AD Kalant H Khanna JM. Effects of treatment dose and intoxicated practice on the development of tolerance to ethanol-induced motor impairment. Alcohol. (1987) Suppl, 1:435–9. 3426712

[26] Tabakoff B Kiianmaa K. Does tolerance develop to the activating, as well as the depressant, effects of ethanol? Pharmacol Biochem Behav. (1982) 17:1073–6. doi: 10.1016/0091-3057(82)90496-8 7178199

[27] Werner DF Swihart AR Ferguson C Lariviere WR Harrison NL Homanics GE. Alcohol-induced tolerance and physical dependence in mice with ethanol insensitive alpha1 GABA A receptors. Alcohol Clin Exp Res. (2009) 33:289–99. doi: 10.1111/j.1530-0277.2008.00832.x PMC2786059 19032579

[28] Matson LM Kasten CR Boehm SL 2nd Grahame NJ. Selectively bred crossed high-alcohol-preferring mice drink to intoxication and develop functional tolerance, but not locomotor sensitization during free-choice ethanol access. Alcohol Clin Exp Res. (2014) 38:267–74. doi: 10.1111/acer.2014.38.issue-1 PMC3844084 23909817

[29] Boehm SL Goldfarb KJ Serio KM Moore EM Linsenbardt DN. Does context influence the duration of locomotor sensitization to ethanol in female DBA/2J mice? Psychopharmacol (Berl). (2008) 197:191–201. doi: 10.1007/s00213-007-1022-6 PMC2279101 18049811

[30] Broadbent J Kampmueller KM Koonse SA. Role of dopamine in behavioral sensitization to ethanol in DBA/2J mice. Alcohol. (2005) 35:137–48. doi: 10.1016/j.alcohol.2005.03.006 15963427

[31] Fletcher PJ Li Z Ji XD Lê AD. Established sensitization of ethanol-induced locomotor activity is not reversed by psilocybin or the 5-HT(2A) receptor agonist TCB-2 in male DBA/2J mice. Pharmacol Biochem Behav. (2024) 235:173703.9. doi: 10.1016/j.pbb.2023.173703 38154589

[32] Melón LC Boehm SL 2nd. Role of genotype in the development of locomotor sensitization to alcohol in adult and adolescent mice: comparison of the DBA/2J and C57BL/6J inbred mouse strains. Alcohol Clin Exp Res. (2011) 35:1351–60. doi: 10.1111/acer.2011.35.issue-7 PMC3117058 21410489

[33] Meyer PJ Phillips TJ. Behavioral sensitization to ethanol does not result in cross-sensitization to NMDA receptor antagonists. Psychopharmacol (Berl). (2007) 195:103–15. doi: 10.1007/s00213-007-0871-3 17653696

[34] Meyer PJ Palmer AA McKinnon CS Phillips TJ. Behavioral sensitization to ethanol is modulated by environmental conditions, but is not associated with cross-sensitization to allopregnanolone or pentobarbital in DBA/2J mice. Neuroscience. (2005) 131:263–73. doi: 10.1016/j.neuroscience.2004.11.005 15708471

[35] Miller CN Kamens HM. Reduced expression of ethanol sensitization by α3β4 nicotinic acetylcholine receptors in DBA/2J mice. Exp Clin Psychopharmacol. (2020) 28:348–54. doi: 10.1037/pha0000324 PMC7117981 31580099

[36] Gallaher EJ Jones GE Belknap JK Crabbe JC. Identification of genetic markers for initial sensitivity and rapid tolerance to ethanol-induced ataxia using quantitative trait locus analysis in BXD recombinant inbred mice. J Pharmacol Exp Ther. (1996) 277:604–12. 8627537

[37] Linsenbardt DN Moore EM Gross CD Goldfarb KJ Blackman LC Boehm SL 2nd. Sensitivity and tolerance to the hypnotic and ataxic effects of ethanol in adolescent and adult C57BL/6J and DBA/2J mice. Alcohol Clin Exp Res. (2009) 33:464–76. doi: 10.1111/j.1530-0277.2008.00857.x PMC2736547 19120054

[38] White A Caillaud M Carper M Poklis J Miles MF Damaj MI. Thermal antinociceptive responses to alcohol in DBA/2J and C57BL/6J inbred male and female mouse strains. Behav Brain Res. (2023) 436:114087. doi: 10.1016/j.bbr.2022.114087 36057379 PMC9999239

[39] Crabbe JC Kosobud A Young ER. Genetic selection for ethanol withdrawal severity: differences in replicate mouse lines. Life Sci. (1983) 33:955–62. doi: 10.1016/0024-3205(83)90751-8 6684200

[40] McClearn GE Kakihana R. Selective breeding for ethanol sensitivity: short-sleep and long-sleep mice. In: McClearn G. E. D., R.A. Erwin VG, editors. Development of Animal Models as Pharmacogenetic Tools. U.S. Government Printing Office, Washington D.C (1981).

[41] Belknap JK Crabbe JC Young ER. Voluntary consumption of ethanol in 15 inbred mouse strains. Psychopharmacol (Berl). (1993) 112:503–10. doi: 10.1007/BF02244901 7871064

[42] Crabbe JC Gallaher ES Phillips TJ Belknap JK. Genetic determinants of sensitivity to ethanol in inbred mice. Behav Neurosci. (1994) 108:186–95. doi: 10.1037/0735-7044.108.1.186 8192844

[43] Gubner NR Wilhelm CJ Phillips TJ Mitchell SH. Strain differences in behavioral inhibition in a Go/No-go task demonstrated using 15 inbred mouse strains. Alcohol Clin Exp Res. (2010) 34:1353–62. doi: 10.1111/j.1530-0277.2010.01219.x PMC3336215 20491731

[44] Logue SF Swartz RJ Wehner JM. Genetic correlation between performance on an appetitive-signaled nosepoke task and voluntary ethanol consumption. Alcohol Clin Exp Res. (1998) 22:1912–20. 9884133

[45] Metten P Crabbe JC. Common genetic determinants of severity of acute withdrawal from ethanol, pentobarbital and diazepam in inbred mice. Behav Pharmacol. (1994) 5:533–47. doi: 10.1097/00008877-199408000-00014 11224305

[46] Metten P Crabbe JC. Alcohol withdrawal severity in inbred mouse (Mus musculus) strains. Behav Neurosci. (2005) 119:911–25. doi: 10.1037/0735-7044.119.4.911 16187819

[47] Lessov CN Phillips TJ. Duration of sensitization to the locomotor stimulant effects of ethanol in mice. Psychopharmacol (Berl). (1998) 135:374–82. doi: 10.1007/s002130050525 9539262

[48] Lessov CN Palmer AA Quick EA Phillips TJ. Voluntary ethanol drinking in C57BL/6J and DBA/2J mice before and after sensitization to the locomotor stimulant effects of ethanol. Psychopharmacol (Berl). (2001) 155:91–9. doi: 10.1007/s002130100699 11374341

[49] National Research Council Committee for the Update of the Guide for the, C Use of Laboratory, A. The National Academies Collection: Reports funded by National Institutes of Health. Guide for the Care and Use of Laboratory Animals. Washington (DC: National Academies Press (US) Copyright © 2011, National Academy of Sciences (2011).

[50] Phillips TJ Feller DJ Crabbe JC. Selected mouse lines, alcohol and behavior. Experientia. (1989) 45:805–27. doi: 10.1007/BF01954056 2570713

[51] Camarini R Pautassi RM. Behavioral sensitization to ethanol: Neural basis and factors that influence its acquisition and expression. Brain Res Bull. (2016) 125:53–78. doi: 10.1016/j.brainresbull.2016.04.006 27093941

[52] Gubner NR Phillips TJ. Effects of nicotine on ethanol-induced locomotor sensitization: A model of neuroadaptation. Behav Brain Res. (2015) 288:26–32. doi: 10.1016/j.bbr.2015.03.066 25857831 PMC4442015

[53] Gubner NR McKinnon CS Phillips TJ. Effects of varenicline on ethanol-induced conditioned place preference, locomotor stimulation, and sensitization. Alcohol Clin Exp Res. (2014) 38:3033–42. doi: 10.1111/acer.12588 PMC4293040 25581658

[54] Harmata GIS Chan AC Merfeld MJ Taugher-Hebl RJ Harijan AK Hardie JB. Intoxicating effects of alcohol depend on acid-sensing ion channels. Neuropsychopharmacology. (2023) 48:806–15. doi: 10.1038/s41386-022-01473-4 PMC10066229 36243771

[55] Kalafateli AL Aranäs C Jerlhag E. Effects of sub-chronic amylin receptor activation on alcohol-induced locomotor stimulation and monoamine levels in mice. Psychopharmacol (Berl). (2020) 237:3249–57. doi: 10.1007/s00213-020-05607-8 PMC7561575 32651639

[56] Palmer AA McKinnon CS Bergstrom HC Phillips TJ. Locomotor activity responses to ethanol, other alcohols, and GABA-A acting compounds in forward- and reverse-selected FAST and SLOW mouse lines. Behav Neurosci. (2002) 116:958–67. doi: 10.1037/0735-7044.116.6.958 12492294

[57] Elvig SK McGinn MA Smith C Arends MA Koob GF Vendruscolo LF. Tolerance to alcohol: A critical yet understudied factor in alcohol addiction. Pharmacol Biochem Behav. (2021) 204:173155. doi: 10.1016/j.pbb.2021.173155 33631255 PMC8917511

[58] Nona CN Hendershot CS Le AD. Behavioral sensitization to alcohol: Bridging the gap between preclinical research and human models. Pharmacol Biochem Behav. (2018) 173:15–26. doi: 10.1016/j.pbb.2018.08.003 30118733

[59] Chesler EJ. Out of the bottleneck: the Diversity Outcross and Collaborative Cross mouse populations in behavioral genetics research. Mamm Genome. (2014) 25:3–11. doi: 10.1007/s00335-013-9492-9 24272351 PMC3916706

[60] Saul MC Philip VM Reinholdt LG Chesler EJ. High-diversity mouse populations for complex traits. Trends Genet. (2019) 35:501–14. doi: 10.1016/j.tig.2019.04.003 PMC6571031 31133439

[61] Solberg Woods LC Palmer AA. Using heterogeneous stocks for fine-mapping genetically complex traits. In: Hayman G Smither J Dwinell M Shimoyama M, editors. Rat Genomics. Methods in Molecular Biology. Humana, New York, NY: Humana Press (2019) 2018:233–47. 10.1007/978-1-4939-9581-3_11 PMC9121584 31228160

[62] Stewart J Badiani A. Tolerance and sensitization to the behavioral effects of drugs. Behav Pharmacol. (1993) 4:289–312. doi: 10.1097/00008877-199308000-00003 11224198

[63] Goudie AJ Emmet-Oglesby MW. Tolerance and sensitization: overview. In: Goudie AJ Emmett-Oglesby ME, editors. Psychoactive Drugs. Contemporary Neuroscience. Humana Press, Totowa, NJ (1989).

[64] Le AD Khanna JM. Dispositional mechanisms in drug tolerance and sensitization. In: Goudie AJ Emmett-Oglesby ME, editors. Psychoactive Drugs. Contemporary Neuroscience. Humana Press, Totowa, NJ (1989).

[65] Pietrzykowski AZ Treistman SN. The molecular basis of tolerance. Alcohol Res Health. (2008) 31:298–309. 23584007 PMC3860466

