# Supplementary material for: Does tolerance to ethanol-induced ataxia explain the sensitized response to ethanol?
Source: Front Psychiatry. 2024 Aug 30;15:1418490. doi: 10.3389/fpsyt.2024.1418490 (PMC11392896; doi:10.3389/fpsyt.2024.1418490)
Supplement: Supplementary file 1 [file DataSheet1.pdf]

Supplementary Material

Does tolerance to ethanol-induced sedation explain the sensitized response to ethanol?

Cheryl Reed[1], Tamara J. Phillips[1,2*]

[1]Department of Behavioral Neuroscience and the Portland Alcohol Research Center, Oregon Health & Science University, Portland, OR, USA
[2]Veterans Affairs Portland Health Care System, Portland, OR, USA

Supplementary Material to Experiment 1: Acute and Repeated EtOH Effects on Locomotor Activity and Coordination in 15 Inbred Mouse Strains.

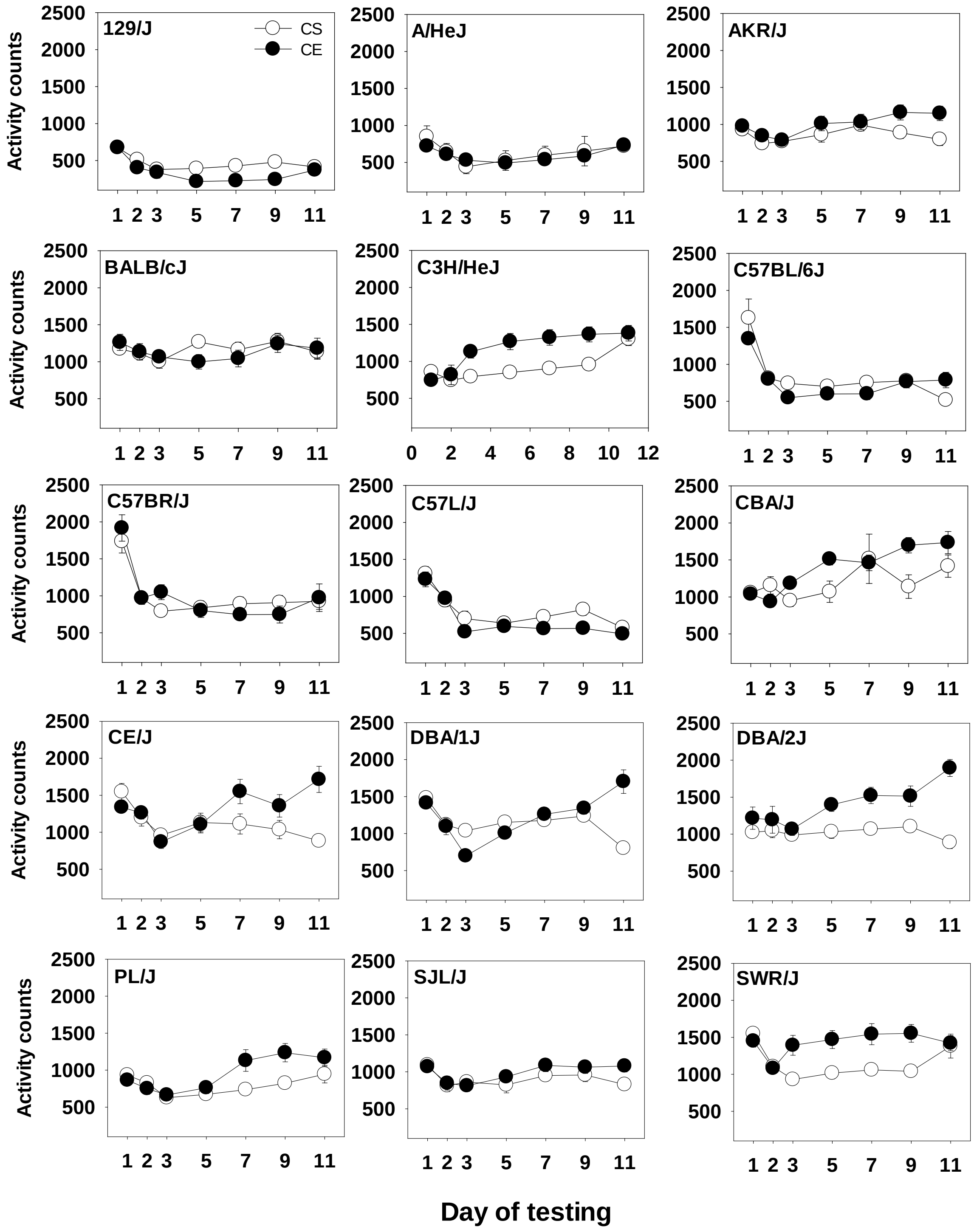

Supplementary Figure S1. Activity counts across days in the chronic saline (CS) and chronic EtOH (CE) groups for each strain. Data shown are means ± SE. N per strain per group = 9-10.

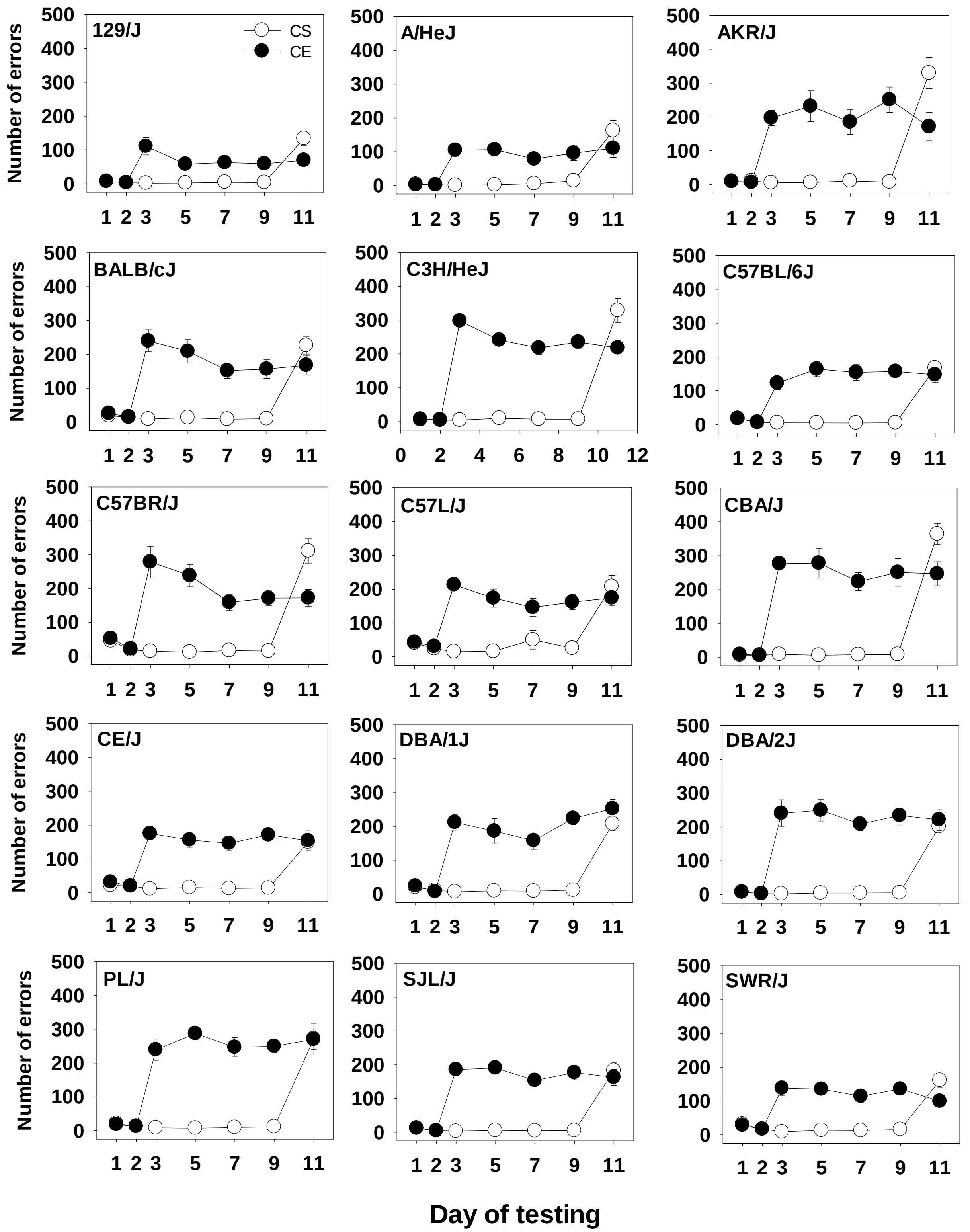

Supplementary Figure S2. Number of errors across days in the chronic saline (CS) and chronic EtOH (CE) groups for each strain. Data shown are means ± SE. N per strain group per group = 9-10.

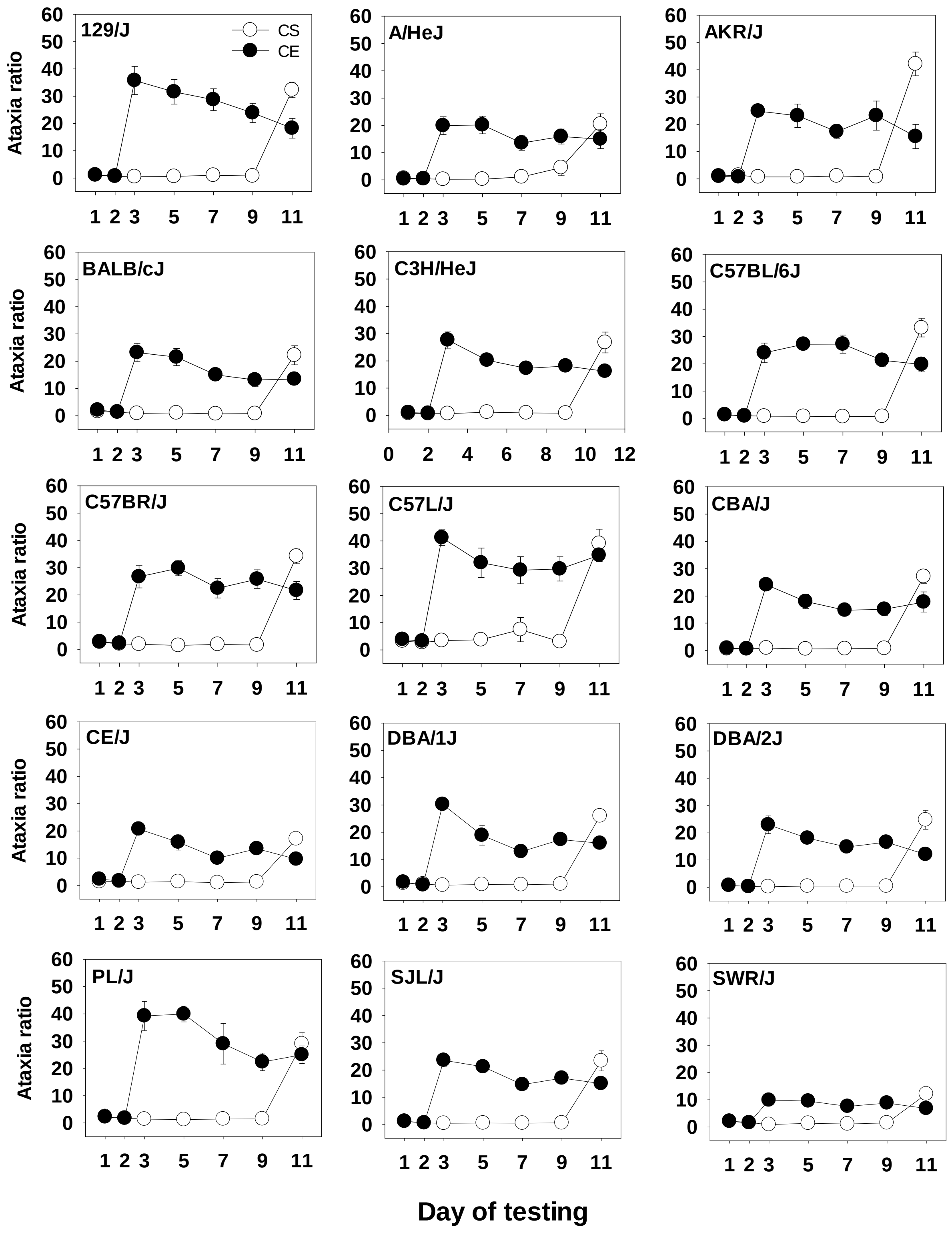

Supplementary Figure S3. Ataxia ratio across days in the chronic saline (CS) and chronic EtOH (CE) groups for each strain. Shown are means ± SE. N per strain per group = 9-10.

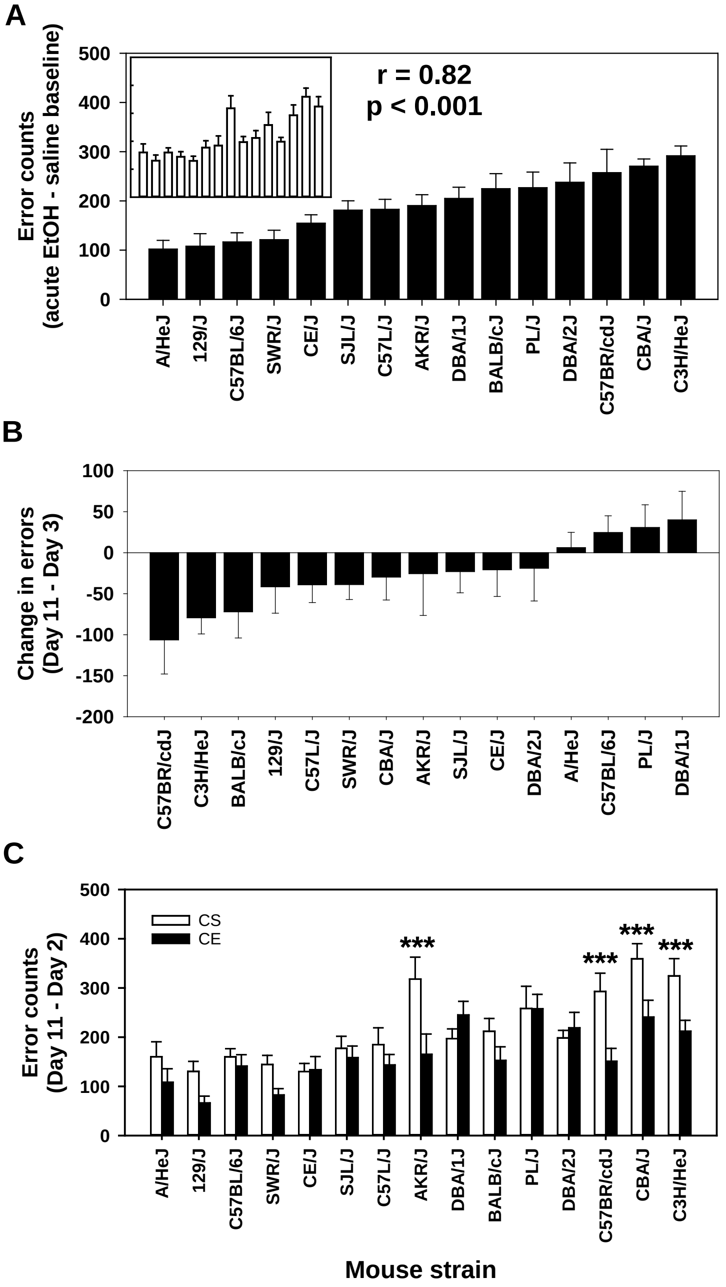

Supplementary Figure S4. Mean EtOH-induced error phenotypes in 15 inbred mouse strains. (A) Solid bars represent day 3 - day 2 error counts for the chronic EtOH (CE) group. Open bars in the inset represent day 11 - day 2 data for the chronic saline (CS) group. Strain order for the CS data is the same as that listed along the x-axis. The r represents the Pearson's correlation between the CE and CS groups for error counts after acute ethanol administration. (B) Change in errors (day 3 – day 3) with repeated ethanol exposure in the CE group. (C) Error counts on day 11 were corrected for baseline errors. Open bars represent the CS group data that received ethanol for the first time on day 11; solid bars represent the CE group data that received EtOH for the fifth time on day 11. Shown are means ± SE. ***$p<0.001$ for the difference between the CS and CE groups within a given strain.

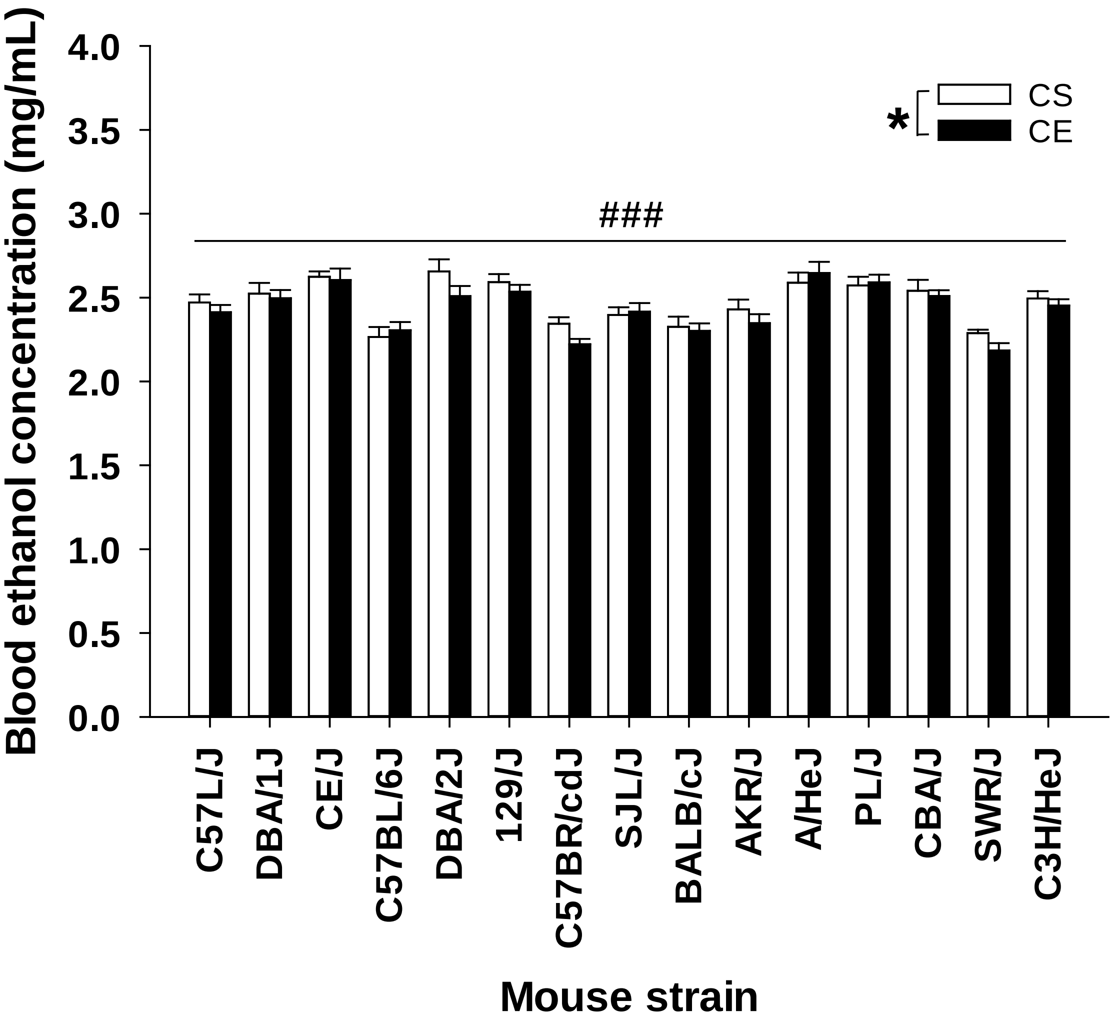

Supplementary Figure S5. Mean blood ethanol concentration (BEC) in 15 inbred mouse strains. Blood samples were collected from mice in experiment 1, immediately following testing on day 11. Solid bars represent BEC (mg/mL) for the chronic EtOH (CE) treatment group, which received EtOH for the fifth time on day 11. Open bars represent BEC for the chronic saline (CS) treatment group which received EtOH for the first time on day 11. Shown are means ± SE. *$p<0.05$ for the main effect of group; ### $p<0.001$ for the main effect of strain.

Analysis of DBA/2J data alone from Experiment 1.

Because we used DBA/2J as a mouse model for high sensitivity to EtOH-induced sensitization, we separately analyzed the DBA/2J strain data from Experiment 1 (Supplementary Figures S6).

Locomotor activity counts. To determine differences between the CS and CE groups within the DBA/2J strain for locomotor activity (Supplementary Figure S6A), repeated measures ANOVA was performed across test days. There was a significant day by treatment group interaction ($F_{[6,108]}=6.8$, $p<0.001$). Further interrogation of this interaction revealed no significant difference in baseline activity (day 2) between the CS and CE groups and no significant difference on day 3 between the CS and CE groups. However, the CE group had higher locomotor activity levels on test days 5, 7, 9 and 11, when compared to the CS group. The difference on day 11 supports between-group sensitization. In the within-group repeated measures ANOVA across days, there was no significant effect of day for the CS group; whereas the effect of day was significant in the CE group ($F_{[6,54]}=7.3$, $p<0.001$). In the CE group, there was greater locomotor activity on day 11 when compared to all other days which supports within-group sensitization. See Supplementary Figure S6A for significant mean differences.

Grid test error counts. To determine differences between the CS and CE groups within the DBA/2J strain for foot slip errors (Supplementary Figure S6B), repeated measures ANOVA was performed across test days. There was a significant day by treatment group interaction ($F_{[6,108]}=28.9$, $p<0.001$). Further interrogation of this interaction revealed no significant difference in baseline errors (day 2) between the groups. There were significantly more foot slip errors in CE than CS group mice on days 3, 5, 7, and 9. On day 11, when CS group mice received EtOH, there was no significant difference in the number of foot slip errors between the CS and CE groups. Repeated measures ANOVA for errors in the CS group found a significant main effect of day ($F_{[6,54]}=160.8$, $p<0.001$), due to a larger number of errors on day 11. Repeated measures ANOVA for errors within the CE group also found a significant main effect of day ($F_{[6,54]}=26.7$, $p<0.001$), with an increase in errors on day 3 compared to baseline, and elevated errors over the course of testing. See Supplementary Figure S6B for significant mean differences.

Grid test ataxia ratio. To determine differences between the CS and CE groups within the DBA/2J strain for ataxia ratio (Supplementary Figure S6C), repeated measures ANOVA was performed across test days. There was a significant day by treatment group interaction ($F_{[6,108]}=37.4$, $p<0.001$). Further interrogation of this interaction revealed no significant difference in baseline ataxia ratio (day 2) between the groups. However, there was significantly greater ataxia in CE compared to CS group mice on days 3, 5, 7, 9 and 11. Repeated measures ANOVA for ataxia ratio in the CS group found a main effects of day ($F_{[6,54]}= 49.5$, $p<0.001$), due to a larger ataxia ratio on day 11. Repeated measures ANOVA for ataxia ratio within the CE group also found a significant main effect of day ($F_{[6,54]}=28.7$, $p<0.001$), with an increase in errors on day 3 decreasing across treatments in support of tolerance. Data are shown in Supplemental Figure 6, panel C. See Supplementary Figure S6C for significant mean differences.

Blood EtOH concentration (BEC). There was no difference between treatment groups for BEC on day 11 in the DBA/2J mice (mean ± SE for CE = 2.51 ± 0.06 mg/ml and CS = 2.66 ± 0.07 mg/ml).

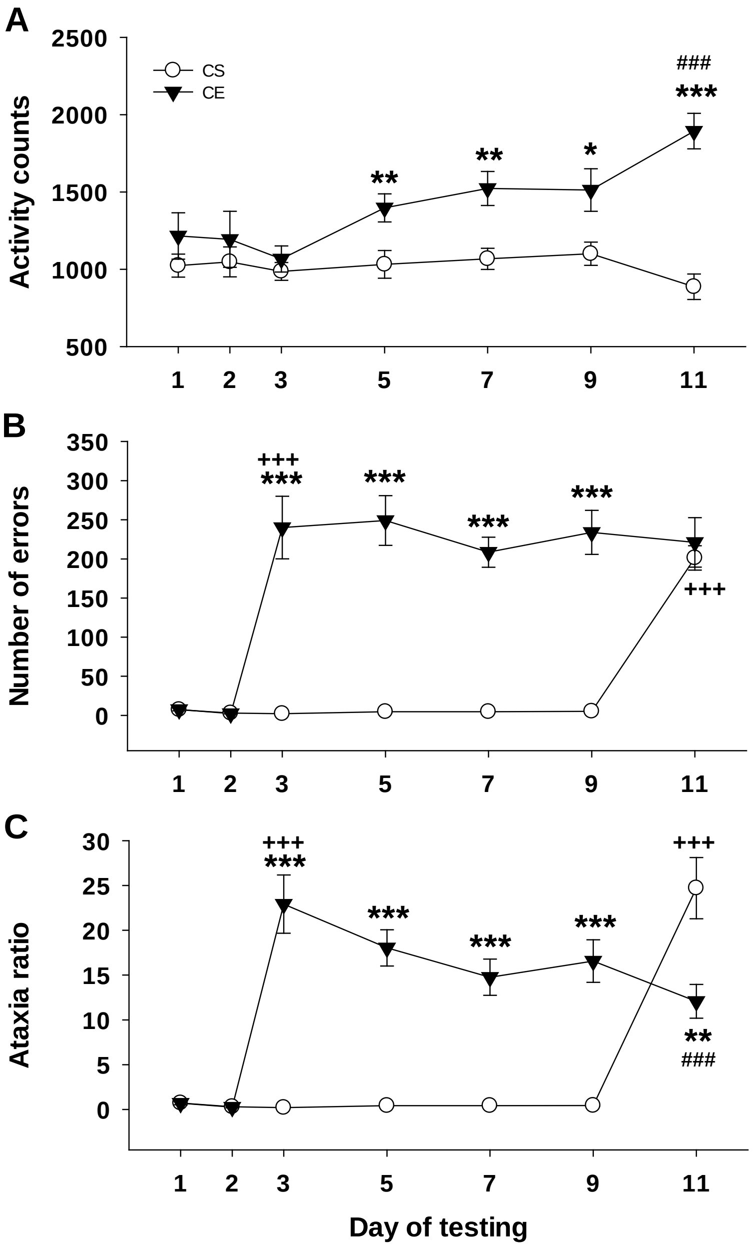

Supplementary Figure S6. Mean locomotor activity, error counts and ataxia ratio for DBA/2J mice from Experiment 1. (A) Mean locomotor activity in CS and CE treatment groups. (B) Mean number of foot slip errors in the CS and CE treatment groups. (C) Mean ataxia ratio in the CS and CE groups. See Table 1 in main manuscript for treatment schedule. Shown are means ± SE. *$p<0.05$, **$p<0.01$, ***$p<0.001$ for the difference between CE and CS groups for that test day; +++$p<0.001$ for the acute response to EtOH (CS group day 11 vs day 2; CE group day 3 vs day 2); ###$p<0.001$ for significant sensitization or tolerance in CE group mice (day 11 vs day 3).

Supplementary Material to Experiments 2, 3 and 4: Uncorrected error counts.

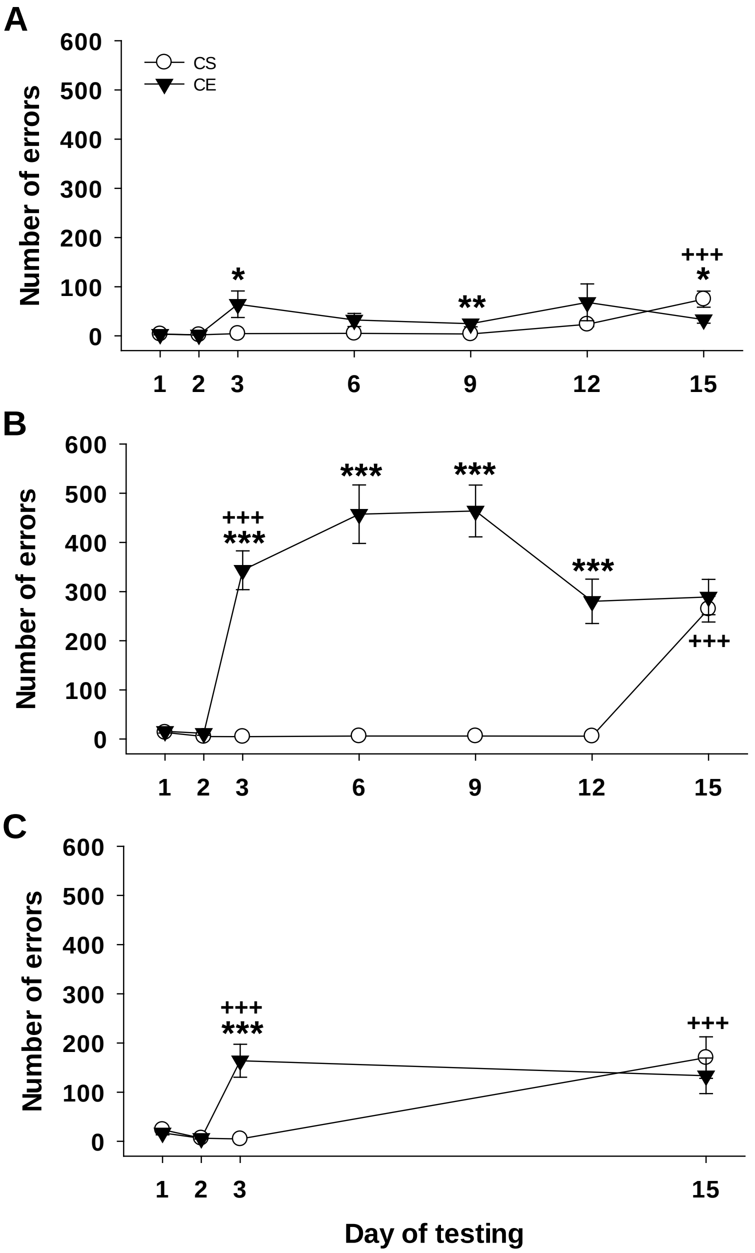

Supplementary Figure S7. Mean uncorrected error count data for mice in experiments 2, 3 and 4 with repeated saline or ethanol administration. (A) Mean errors for CS and CE treatment groups from Experiment 2; DBA/2J mice. (B) Mean errors for CS and CE treatment groups from Experiment 3; WSC mice (C) Mean errors for CS and CE treatment groups for Experiment 4; WSC mice with limited exposure to grid test. Shown are means ± SE. *p<0.05, **p<0.1 ***p<0.001 for the difference between CE and CS groups for that test day; +++p<0.001 for the acute response to EtOH (CS group day 15 vs day 2; CE group day 3 vs day 2).
